# Identification of biomarkers to stratify response to B-cell-targeted therapies in systemic lupus erythematosus: an exploratory analysis of a randomised controlled trial

**DOI:** 10.1016/S2665-9913(22)00332-0

**Published:** 2022-11-28

**Authors:** Muhammad Shipa, Liliana R Santos, Dao X Nguyen, Andrew Embleton-Thirsk, Mariea Parvaz, Lauren L Heptinstall, Ruth J Pepper, David A Isenberg, Caroline Gordon, Michael R Ehrenstein

**Affiliations:** a Department of Rheumatology, University College London, London, UK; b Comprehensive Clinical Trials Unit, University College London, London, UK; c Department of Renal Medicine, Royal Free Hospital, University College London, London, UK; d Rheumatology Research Group, Institute of Inflammation and Ageing, University of Birmingham, Birmingham, UK

## Abstract

**Background:**

Systemic lupus erythematosus (SLE) is a complex autoimmune disease associated with widespread immune dysregulation and diverse clinical features. Immune abnormalities might be differentially associated with specific organ involvement or response to targeted therapies. We aimed to identify biomarkers of response to belimumab after rituximab to facilitate a personalised approach to therapy.

**Methods:**

In this exploratory analysis of a randomised controlled trial (BEAT-LUPUS), we investigated immune profiles of patients with SLE recruited to the 52-week clinical trial, which tested the combination of rituximab plus belimumab versus rituximab plus placebo. We used machine learning and conventional statistics to investigate relevant laboratory and clinical biomarkers associated with major clinical response. BEAT LUPUS is registered at ISRCTN, 47873003, and is now complete.

**Findings:**

Between Feb 2, 2017, and March 28, 2019, 52 patients were recruited to BEAT-LUPUS, of whom 44 provided clinical data at week 52 and were included in this analysis. 21 (48%) of 44 participants were in the belimumab group (mean age 39·5 years [SD 12·1]; 17 [81%] were female, four [19%] were male, 13 [62%] were White) and 23 (52%) were in the placebo group (mean age 42·1 years [SD 10·5]; 21 [91%] were female, two [9%] were male, 16 [70%] were White). Ten (48%) of 21 participants who received belimumab after rituximab and eight (35%) of 23 who received placebo after rituximab had a major clinical response at 52 weeks (between-group difference of 13% [95% CI –15 to 38]). We found a predictive association between baseline serum IgA2 anti-double stranded DNA (dsDNA) antibody concentrations and clinical response to belimumab after rituximab, with a between-group difference in major clinical response of 48% (95% CI 10 to 70) in patients with elevated baseline serum IgA2 anti-dsDNA antibody concentrations. Moreover, among those who had a major clinical response, serum IgA2 anti-dsDNA antibody concentrations significantly decreased from baseline only in the belimumab group. Increased circulating IgA2 (but not total) plasmablast numbers, and T follicular helper cell numbers predicted clinical response and were both reduced only in patients who responded to belimumab after rituximab. Serum IgA2 anti-dsDNA antibody concentrations were also associated with active renal disease, whereas serum IgA1 anti-dsDNA antibody and IFN-α concentrations were associated with mucocutaneous disease activity but did not predict response to B-cell targeted therapy. Patients with a high baseline serum interleukin-6 concentration were less likely to have a major clinical response, irrespective of therapy.

**Interpretation:**

This exploratory study revealed the presence of distinct molecular networks associated with renal and mucocutaneous involvement, and response to B-cell-targeted therapies, which, if confirmed, could guide precision targeting of advanced therapies for this heterogenous disease.

**Funding:**

Versus Arthritis, UCLH Biomedical Research Centre, LUPUS UK, and GSK.

## Introduction

Systemic lupus erythematosus (SLE) is an autoimmune disease characterised by an array of clinical features and immune abnormalities. Molecular and immunological phenotyping has stratified patients with SLE into several major groups, which contributes to the heterogeneous clinical presentation, severity, and clinical outcomes, and might explain the highly variable and moderate responses to targeted therapies.^1^

The production of autoantibodies against nuclear proteins, in particular double-stranded DNA (dsDNA), is a hallmark of SLE and provides a persuasive rationale for use of B-cell-targeted therapies, such as rituximab.^2^ However, considerable variation in response to rituximab has been noted, and randomised clinical trials have not shown an overall benefit. The B-cell-activating factor (BAFF; also known as B-lymphocyte stimulator, or BLyS)-neutralising monoclonal antibody belimumab was the first biologic licensed for the treatment of SLE.^3^ Increased concentrations of BAFF and the association between increased BAFF concentrations and worsening disease after rituximab therapy,^4^ led us to design a placebo-controlled clinical trial (BEAT-LUPUS) comparing treatment with belimumab after rituximab to rituximab alone for patients with SLE whose disease was refractory to conventional therapy. The BEAT-LUPUS trial showed that the combination of belimumab after rituximab significantly reduced serum IgG anti-dsDNA antibody concentrations and the risk of severe flares compared with rituximab alone during the 52 weeks of treatment.^5^

**Research in context**

**Evidence before this study**

There is an urgent need for biomarkers that can inform a personalised medicine approach and improve on the modest results of clinical trials testing targeted therapies for patients with systemic lupus erythematosus (SLE). Moderate efficacy coupled with high costs of drugs leads to restricted or no access to licensed advanced therapies for patients in many countries, such as England, UK. Computational methods based on unsupervised machine learning have the potential to stratify patients with SLE to identify distinct molecular endotypes that are most likely to respond to a targeted therapy. The BEAT-LUPUS placebo-controlled trial tested the combination of belimumab after rituximab compared with rituximab followed by placebo in 52 patients with SLE whose disease was refractory to conventional therapy. Belimumab after rituximab significantly reduced IgG anti-double stranded DNA (dsDNA) antibodies and the incidence of severe flares compared with placebo. We searched PubMed, Web of Science, and Google Scholar for research articles, in English, published between Jan 1, 1990, and June 30, 2022, using the terms “systemic lupus erythematosus”, “biomarkers”, “rituximab”, and “belimumab”. We identified no studies that identified biomarkers that predicted response to belimumab after rituximab in patients with SLE, but increased serum IgG anti-dsDNA antibodies and low complement component C3 were associated with active SLE and have been identified as potential biomarkers of response to belimumab alone.

**Added value of this study**

By applying machine learning to the immune and clinical profiles of patients recruited to the BEAT-LUPUS trial, distinct immunological and molecular networks were identified that were associated with response to combination belimumab after rituximab, and with specific organ involvement in SLE, that could guide precision targeting of specific therapies. Serum IgA2 anti-dsDNA antibody concentrations emerged as the only predictive biomarker of response to belimumab after rituximab, and when used as an effect modifier of treatment response, we found a substantially increased difference in treatment outcome (major clinical response) between the belimumab and placebo group (both after rituximab) compared with the trial cohort analysed without use of a biomarker.

**Implications of all the available evidence**

Serum IgA2 anti-dsDNA antibody concentration is a technically simple biomarker to assay and could be incorporated into routine clinical practice to guide patient selection and improve access to rituximab followed by belimumab combination therapy for patients with SLE. IgA1 anti-dsDNA antibodies could be used to monitor skin disease activity and IgA2 anti-dsDNA antibodies to monitor renal disease activity in SLE. Further validation of our findings in a larger trial is required.

Stratification of patients is likely to aid treatment selection and improve outcomes, given the immunopathological and clinical complexity of SLE combined with the variable response to targeted therapy. Using samples from patients recruited to the BEAT-LUPUS trial, we aimed to identify biomarkers of response to belimumab after rituximab to aid a personalised approach to therapy and provide insights into mechanisms of action that could lead to development of further novel therapeutic strategies.

## Methods

### Study design and participants

BEAT-LUPUS was a 52-week, multicentre, randomised, double-blind, placebo-controlled, parallel group, phase 2b clinical trial, based in the UK, investigating efficacy of belimumab starting 4–8 weeks after the first infusion of B-cell-depleting therapy (rituximab) in patients with SLE.^5^, ^6^ Briefly, patients with SLE, aged 18–75 years, who were refractory to conventional treatment, had a positive anti-dsDNA antibody test at least once in the 5 years before randomisation, and whose treating physicians had recommended rituximab, were recruited from 16 hospitals across England.

As part of this exploratory analysis, additional stored serum samples from two independent cohorts of patients with SLE were also analysed: one cross-sectional cohort with active renal or mucocutaneous disease (organ involvement validation cohort) and a second cohort treated with belimumab alone (belimumab only cohort), as part of standard of care in accordance with UK National institute for Heath and Care Excellence policy before December 2021, with samples taken before and after treatment. Renal biopsy samples from five patients with lupus nephritis who were known to have glomerular IgA deposits were analysed for glomerular IgA1 and IgA2 deposition. Serum samples from healthy volunteer donors recruited from among University College London (UCL; London, UK) staff were used to define the upper limit of normal for all in-house ELISA assays.

The Hampstead Research Ethics Committee-London approved the BEAT-LUPUS trial protocol (reference 16/LO/1024) and the analysis of the additional cohorts and healthy controls (reference 13/LO/0999). The study was conducted in accordance with the principles of the Declaration of Helsinki Good Clinical Practice guidelines. All participants provided written informed consent before enrolment.

### Procedures

Blood samples provided from the BEAT-LUPUS trial were analysed at University College London (UCL; London, UK). We used commercially available ELISA kits to assess IgG anti-dsDNA antibodies (ELISA kit [KA 1100], Abnova, Taoyuan City, Taiwan), IgM anti-dsDNA antibodies (ELISA kit [KA 1099 IgM], Abnova), and IgA anti-dsDNA antibodies (ELISA kit [KA 1198], Abnova), IgG extractable nuclear antigen (ENA) antibodies (KA 1103 [ENA Combi ELISA kit], Abnova), and serum BAFF (DY124-05 [BAFF/BLyS/TNFSF13B DuoSet ELISA] R&D systems, Minneapolis, MN, USA). We used in-house kits to assess IgE, IgG1, IgG2, IgG3, IgG4, IgA1, and IgA2 anti-dsDNA antibodies, and IgA1 and IgA2 ENA-antibodies; additional methods are in the appendix (p 1). For cytokines, we used Simoa Human Cytokine 6-Plex Panel-1 Advantage Kit (Quanterix, Billerica, MA, USA) to measure serum concentrations of IFN-γ, interleukin (IL)-10, IL-12p70, IL-17A, IL-6, and tumour necrosis factor (TNF), and to measure IFN-α we used the Simoa IFN-α Advantage Kit (100860, Quanterix, Billerica, MA, USA) on a Simoa HD-1 analyser. We used expression of *ISG15*, *IFI44*, and *RASD2* genes to determine the IFN-A score and expression of *STAT1*, *SERPING1, BST2,* and *SP100* genes to determine the IFN-B score, adapted from the study by El-Sherbiny and colleagues.^6^, ^7^ We did immune cell phenotyping using an LSR2 or Fortessa flow cytometer (BD Biosciences, Franklin Lakes, NJ, USA) and Diva software (version 9.0.0), and analysed with FlowJo (version 10.7.1; BD Bioscences). Further details of procedures are in the appendix (pp 1–3).

### Outcomes

The principal clinical outcomes used for this exploratory analysis was major clinical response, which was defined as a reduction in British Isles lupus assessment group-2004 (BILAG-2004) index score of A or B, to a score of C or D, and a score that remained at E in other domains, a reduction in daily steroid dose to 7·5 mg or lower, and a modified Systemic Lupus Erythematosus Disease Activity Index 2000 (SLEDAI-2K) score of 2 or lower (without including the anti-dsDNA antibody component).^8^ Renal response was defined as no BILAG-2004 index A or B scores in the renal domain, daily steroid dose of 7·5 mg or lower, urine protein-to-creatinine ratio of 50 mg/mmol or lower or a urine albumin-to-creatinine ratio of 50 mg/mmol or lower, no active urine sediment, and estimated glomerular filtration rate (eGFR) of 60 mL/min per 1·72 m^2^ or higher (or if eGFR was ≤60 mL/min per 1·72 m^2^ at baseline, had not decreased by ≥20%).^8^, ^9^ Mucocutaneous response was defined as no BILAG-2004 index A or B score in the mucocutaneous domain, daily steroid dose of 7·5 mg or lower, and modified SLEDAI-2K of 2 or lower.

Active renal disease was defined as a BILAG-2004 index A/B score in the renal domain at screening with an urine protein-to-creatinine ratio of more than 50 mg/mmol, or urine albumin-to-creatinine ratio of more than 50 mg/mmol, or active urinary sediment with urine protein-to-creatinine ratio of more than 25 mg/mmol at baseline.^8^, ^9^ Active mucocutaneous disease was defined as a BILAG-2004 score of A or B in the mucocutaneous domain.

Severe disease flare was defined as one or more BILAG-2004 A flares and moderate flare as two or more BILAG-2004 B flares but no A flare; flares required worsening or new manifestations of lupus measured using the BILAG-2004 index.^10^

### Statistical analysis

We report demographic data as mean (SD) or median (IQR), depending on the distribution. For continuous variables, we analysed comparison between the two groups using either Student's *t* test (parametric) or the Mann-Whitney *U* test (non-parametric) or for non-parametric pairwise comparison, we used Dunn's multiple comparison test with Bonferroni's adjustment (non-parametric). To select important predictive variables, we applied the supervised machine learning approaches of sparse Partial Least Squares Discriminant Analysis (sPLS-DA) and Regularised Random-Forest (RRF). We report odds ratios (ORs) with 95% CIs for estimates of prediction of response or prediction of active organ involvement. We constructed the final model using variables selected using a random forest (RF) algorithm^11^ using a conventional multiple logistic regression model. We used Kaplan-Meier curves for the time to flare analysis, and we used unadjusted log-rank tests to assess between-group differences. We estimated hazards ratios (HRs) of flares using Cox regression analysis. For longitudinal changes, we fitted a linear or generalised linear mixed-effect model to estimate the mean change with fixed effect of treatment group or treatment response intercepting with trial timepoints from randomisation to 52 weeks, and included a random patient effect to account for clustering by patient, and adjusted for screening value, age, sex, concomitant mycophenolate (yes *vs* no), and prednisolone dose. We applied the bootstrapping resampling approach for multiple testing correction.^12^ For associations with organ involvement, we used the non-parametric Spearman's rank test. We used a p value threshold of 5% for significance to ensure that the probability of a type 1 error did not exceed 5%. Additional details of the statistical analysis are in the appendix (pp 3–5).

We did all statistical analysis using R (version 4.0.2) for Mac OS. Two-sided p values and 95% CIs are reported for all statistical tests. BEAT LUPUS is registered with ISRCTN, 47873003.

### Role of the funding source

The funders had no role in the study design, data collection, data analysis, data interpretation, or writing of the report.

## Results

Between Feb 2, 2017, and March 28, 2019, 52 participants were enrolled in the BEAT-LUPUS trial,^5^ of whom 44 (85%) provided clinical data at 52 weeks and were included in the response prediction models (appendix p 7). 21 (48%) of 44 participants were in the belimumab group (mean age 39·5 years [SD 12·1]; 17 [81%] were female, four [19%] were male, 13 [62%] were White, three [14%] were Black, two [10%] were south Asian, two [10%] were other ethnicity, and one [5%] was Chinese) and 23 were in the placebo group (mean age 42·1 years [SD 10·5]; 21 [91%] were female, two [9%] were male, 16 [70%] were White, two [9%] were Black, one [4%] was south Asian, one [4%] was Chinese, and three [13%] were other ethnicities; appendix pp 8–10). Demographics of the independent organ involvement validation cohort and the belimumab only cohort are shown in the appendix (pp 11–12).

**Figure 1:**
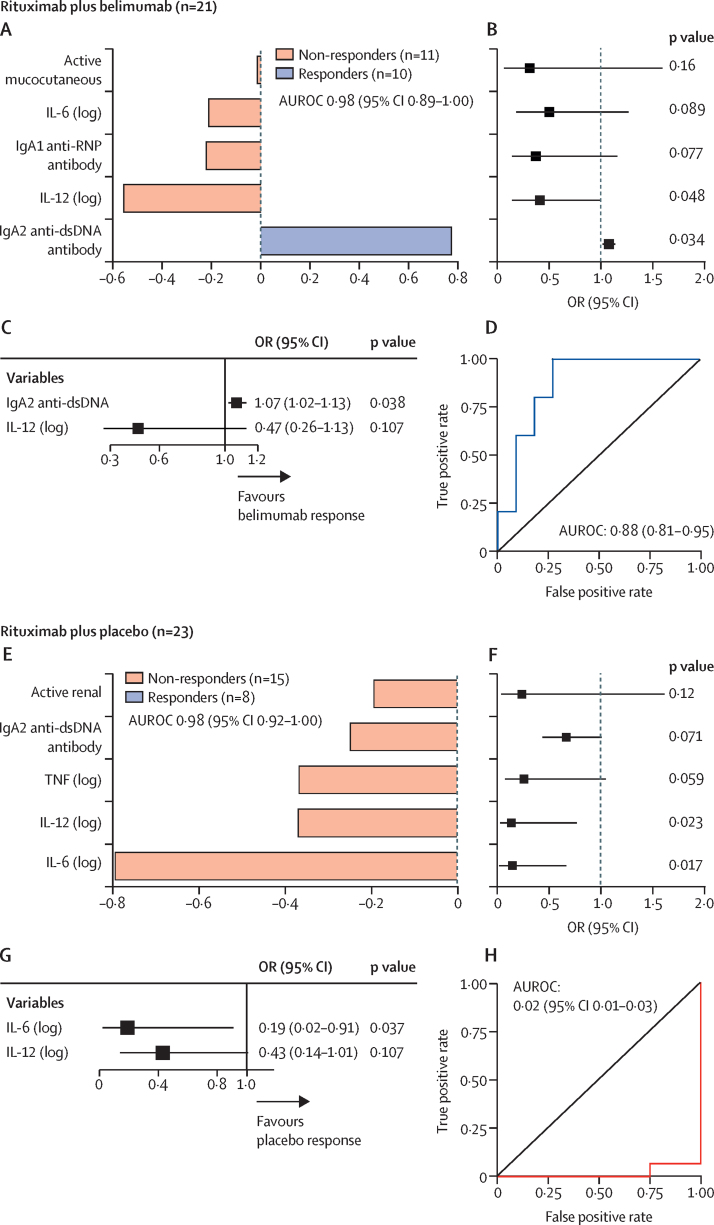
Baseline predictors of major clinical response to belimumab (A–D) and placebo (E–H; both after rituximab), at 52 weeks in the BEAT-LUPUS trial (A, E) Sparse Partial Least Squares Discriminant Analysis (sPLS-DA); factor-loading weights in component 1 are shown for the top five ranked parameters, as chosen by model optimisation, to predict major clinical response at 52 weeks for belimumab (A) and placebo (E) groups. (B, F) Forest plot showing the OR and 95% CI, calculated via univariate logistic regression, of the variables chosen by sPLS-DA with p values for belimumab (B) and placebo (F) groups. (C, G) Results of multiple logistic regression analysis to construct the final model to predict belimumab (C) or placebo (G) response at 52 weeks, in which variables were selected by random forest classification algorithm; with AUROC of this final model to predict belimumab (D) and placebo (H) response. Unit changes for the continuous variables used in the logistic regression are shown in the appendix (p 6). AUROC=area under the receiver operator curve. ds-DNA=double-strand DNA.

Ten (48%) of 21 participants who received belimumab after rituximab and eight (35%) of 23 who received placebo plus rituximab had a major clinical response at 52 weeks (between-group difference of 13% [95% CI –15 to 38]). We constructed a model using a range of clinical and laboratory data (appendix p 6) to identify variables at baseline (screening) that could predict major clinical response at 52 weeks in both groups of the trial. Baseline serum IgA2 anti-dsDNA antibody concentrations emerged as the most important variable in predicting major clinical response in patients treated with belimumab (figure 1A), and yielded the only positive OR (1·08 [95% CI 1·02–1·14]; p=0·034, for each arbitrary unit [AU] change) of the top five variables identified (figure 1B). The imputed ORs of the univariate model are shown in figure 1B and the corresponding complete case analysis in the appendix (pp 13–14). A sensitivity analysis using RRF confirmed that serum IgA2 anti-dsDNA antibody concentration was the most influential variable to predict major clinical response to belimumab at 52 weeks (appendix pp 13–14). Serum IgA2 anti-dsDNA antibody and IL-12 concentrations were selected by random forest^11^, ^13^ for the final multiple logistic regression model (figure 1C; complete case analysis is in the appendix [pp 13–14]). In patients treated with belimumab, each AU increase of serum IgA2 anti-dsDNA antibody at baseline resulted in an OR of 1·07 (95% CI 1·02–1·13]; p=0·038) for attaining major clinical response at 52 weeks (figure 1C). The area under the receiver operator characteristic curve (AUROC) of this final model in predicting major clinical response was 0·88 (95% CI 0·81–0·95). By contrast, we found that the top five baseline variables selected by sPLS-DA from patients in the placebo group of the trial were associated with an unfavourable outcome at 52 weeks (figure 1E). Serum IL-6 (log-transformed) concentration was the strongest negative predictor of attaining a major clinical response at 52 weeks (OR 0·15 [95% CI 0·05–0·67]; p=0·017), followed by serum IL-12 (log-transformed) and IgA2 anti-dsDNA antibody concentrations (figure 1F). Variables with missing values showed similar ORs by complete-case analysis (appendix pp 13–14). Selection of variables by random forest verified the sensitivity of these variables in predicting major clinical response and confirmed the negative association of IL-6 (appendix pp 13–14). In the final multivariate model, the OR of serum IL-6 (log-transformed) as a predictor of major clinical response at 52 weeks was 0·19 (95% CI 0·02–0·91; p=0·037; figure 1G; complete-case analysis is in the appendix [pp 13–14]). The AUROC of the final model in predicting major clinical response in patients treated with placebo was 0·02 (95% CI 0·01–0·03). A direct comparison of these three variables stratified by major clinical response is shown in the appendix (pp 13–14).

**Figure 2:**
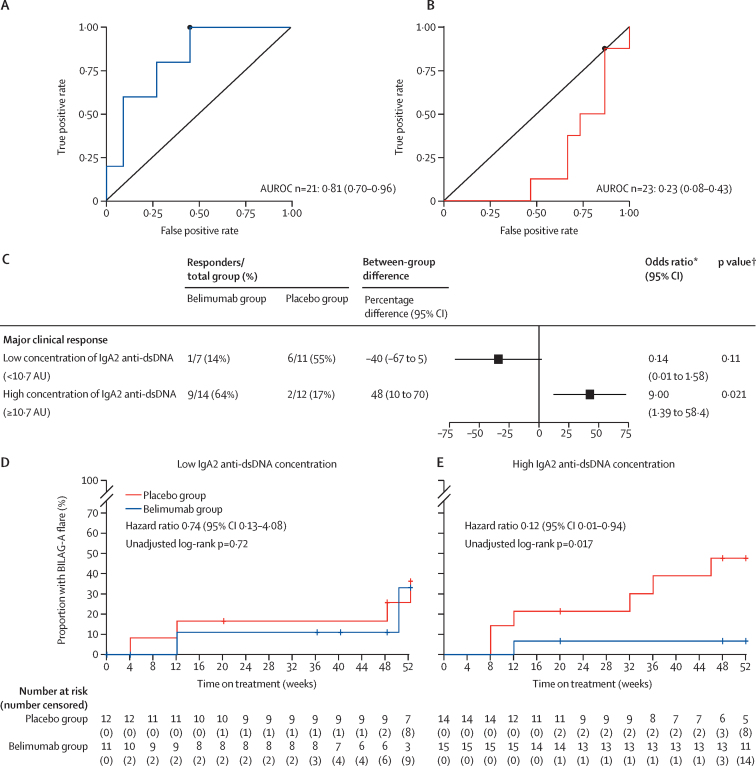
Baseline serum IgA2 anti-dsDNA antibody concentrations as a predictor of clinical response (A–C) and severe flares D, E) in the BEAT-LUPUS trial (A, B) AUROC of serum IgA2 anti-dsDNA antibodies at baseline to predict treatment response to belimumab (A) and placebo (B) at 52 weeks. (C) Serum IgA2 anti-dsDNA antibody concentration (patients were categorised into high or low concentration groups based on the optimal cut-off point from the AUROC analysis in part A, which was 10·7 AUs) was tested as an effect modifier of clinical response at 52 weeks. (D,E) Occurrence of severe flares stratified by low (D) and high (E) serum IgA2-anti-dsDNA antibody concentrations. AU=arbitrary unit. AUROC=area under the receiver operator characteristic curve. dsDNA=double-stranded DNA. *Odds ratios with 95% CIs are provided to predict major clinical response and calculated with logistic regression. †Calculated using Fisher's exact test.

Baseline serum IgA2 anti-dsDNA antibody concentrations alone predicted major clinical response in patients treated with belimumab (AUROC 0·81 [95% CI 0·70–0·96]) but negatively predicted a major clinical response in the placebo group (0·23 [0·08–0·43]; figure 2A, B). The optimal cutoff point for serum IgA2 anti-dsDNA antibody concentrations to predict response in the belimumab group was 10·7 AUs (95% CI 7·8–16·2), with a sensitivity of 1·00 (95% CI 0·81–1·00), a specificity of 0·55 (95% CI 0·45–0·77), and a number need to treat of 2·1 (95% CI 1·2–6·7). We next compared the clinical outcome with respect to major clinical response in both groups of the BEAT-LUPUS trial categorising participants according to whether they had high or low serum concentrations of IgA2 anti-dsDNA antibodies using the cutoff point derived from the AUROC analysis. Patients with high IgA2 anti-dsDNA antibody concentrations at baseline were more likely to have a major clinical response when treated with belimumab after rituximab than they were when given placebo (ie, rituximab alone; between-group difference of 48% [95% CI 10–70]; p=0·021; figure 2C) compared with a between-group difference of 13% [95% CI –15 to 38] without this biomarker. Similarly, the reduction in the risk of a severe flare (BILAG-2004 grade A) with belimumab compared with placebo^5^ was more substantial in participants with a high serum IgA2 anti-dsDNA antibody concentration at baseline than in those with a low concentration at baseline (figure 2D, E). The reduction in the risk of moderate and severe flares was greater with belimumab than with placebo in patients with high serum IgA2 anti-dsDNA antibody concentrations; for those with low serum IgA2 anti-dsDNA concentrations, no difference was seen between the treatment groups (appendix p 15).

**Figure 3:**
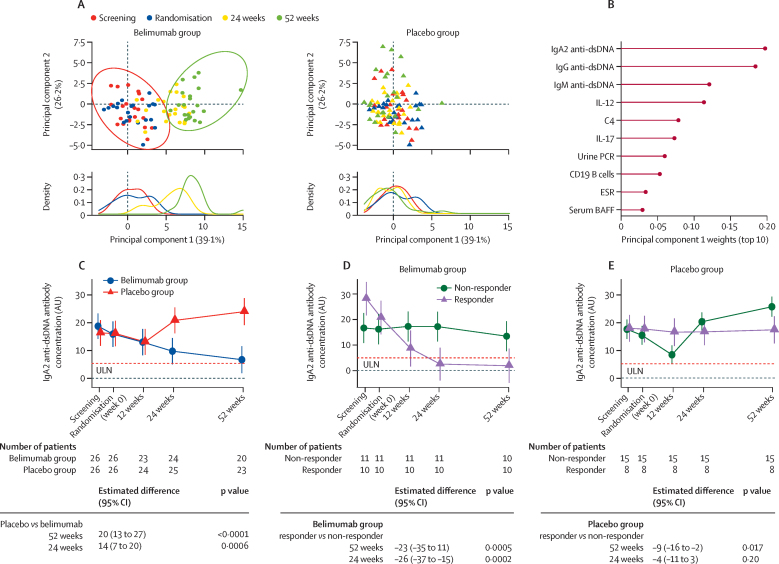
Change in serum IgA2 anti-dsDNA antibody concentrations with belimumab after rituximab versus placebo after rituximab in patients with SLE (A) Principal component analysis of variables (listed in the appendix [p 6]) was done from baseline through to 52 weeks and split into treatment groups and timepoints for visualisation purposes. Each datapoint in the top panel represents a patient, and in the bottom panel shows population densities stratified by treatment and timepoint. The first two principal components described 65·3% of the variance. (B) The contribution of the top 10 variables loading weights in principal component 1. (C–E) Longitudinal change of serum IgA2 anti-dsDNA antibodies stratified by treatment (ie, belimumab *vs* placebo [after rituximab]; C), treatment response in belimumab treated group (D), and treatment response in placebo treated group (E). A longitudinal linear mixed-effect model was fitted with random patient effect to account for clustering by patients and fixed effect of treatment group intercepting with trial times and adjusted for screening IgA2 anti-dsDNA antibody concentrations, age, sex, concomitant mycophenolate (yes or no), and prednisolone dose at respective timepoints to calculate expected difference at 24 and 52 weeks in serum IgA2 anti-dsDNA antibodies. Estimated mean with 95% CIs and number of patients at each timepoints (n) are shown; p values at weeks 24 and 52 are provided. Horizontal dotted line indicates the ULN (3 SDs above the mean of healthy control samples). AU=arbitrary unit. ESR=erythrocyte sedimentations rate. dsDNA=double stranded DNA. PCR=protein creatinine ratio. ULN=upper limit of normal.

Having identified variables at baseline that could predict major clinical response at 52 weeks, we did a principal component analysis (PCA) to interrogate variance between belimumab and placebo during the course of the trial, irrespective of clinical response. Variables measured in samples from screening, randomisation, and 24 and 52 weeks were included (appendix p 6). The highest proportion of variance was explained by belimumab-associated changes with distinct separation at 52 weeks from baseline, which was not found for placebo (figure 3A). Serum IgA2 anti-dsDNA antibody concentration was the top-ranked contributor to this separation (figure 3B). Belimumab significantly reduced serum IgA2 anti-dsDNA antibody concentration compared with placebo at 24 weeks (estimated difference of 14 AUs [95% CI 7–20]; p=0·0006) and 52 weeks (20 AUs [13–27]; p<0·0001; figure 3C). Analysis by clinical response showed that serum IgA2 anti-dsDNA antibody concentration decreased only in patients treated with belimumab who had a major clinical response, decreasing by 60% from baseline at 52 weeks (estimated difference at 52 weeks between responders and non-responders: –23 AUs [–35 to –11]; p=0·0005; figure 3D). There was no such reduction from baseline to 52 weeks in serum IgA2 anti-DNA antibody concentration in the placebo group (figure 3E), although non-responders in the placebo group had an increase in serum IgA2 anti-dsDNA antibody concentration from baseline to 52 weeks (estimated difference at 52 weeks between responders and non-responders: –9 AUs [95% CI –16 to –2]; p=0·017). At baseline, 20 (77%) of 26 patients in the belimumab group and 22 (85%) of 26 in the placebo group were positive for serum IgA2 anti-dsDNA antibodies, which reduced to seven (27%) at 52 weeks in the belimumab group, but remained little changed (21 [81%]) in the placebo group. The OR for reversion to seronegativity for IgA2 anti-dsDNA antibodies at 52 weeks in the belimumab group compared with placebo was 9·6 (95% CI 2·2–42·4; p=0·0033; appendix p 16). By contrast, the proportion of patients who remained IgG anti-dsDNA antibody positive at 52 weeks were similar between the belimumab and placebo groups (appendix p 16). Total serum IgA2 concentrations were decreased in patients treated with belimumab, but did not differ in patients according to major clinical response (appendix p 17). Serum IgA1 anti-dsDNA antibody concentrations were not affected by treatment or clinical response (appendix p 18). We have previously shown a significant reduction in serum IgG anti-dsDNA antibody concentrations in patients treated with belimumab compared with those treated with placebo after rituximab.^5^ However, no significant differences in serum IgG anti-dsDNA antibody concentrations were observed at 24 or 52 weeks between belimumab responders and non-responders (appendix p 19). Serum IgG anti-dsDNA antibody concentrations were lower in responders than in non-responders in the placebo group at 52 weeks (estimated difference of 0·91 [95% CI 0·52–1·30]; p=0·0005; appendix p 19). Serum IgM anti-dsDNA antibody concentrations at 52 weeks were decreased in the placebo group compared with the belimumab group where there was an increase from baseline (estimated difference –82·8 IU/mL [95% CI –109 to –56·8]; p=0·0004; appendix p 20). Serum IgM anti-dsDNA antibody concentrations at 24 and 52 weeks were increased in responders compared with non-responders, particularly in the belimumab group (appendix p 20). To determine whether the reduction in serum IgA2 anti-dsDNA antibody concentrations occurred with belimumab therapy without pre-treatment with rituximab, we analysed serum samples from an independent cohort of 21 patients with SLE before and after belimumab as part of their standard of care in accordance with NHS restrictions related to the use of belimumab for SLE. Serum IgA2 anti-dsDNA antibody concentrations were not affected by belimumab alone compared with combination therapy (appendix p 21).

**Figure 4:**
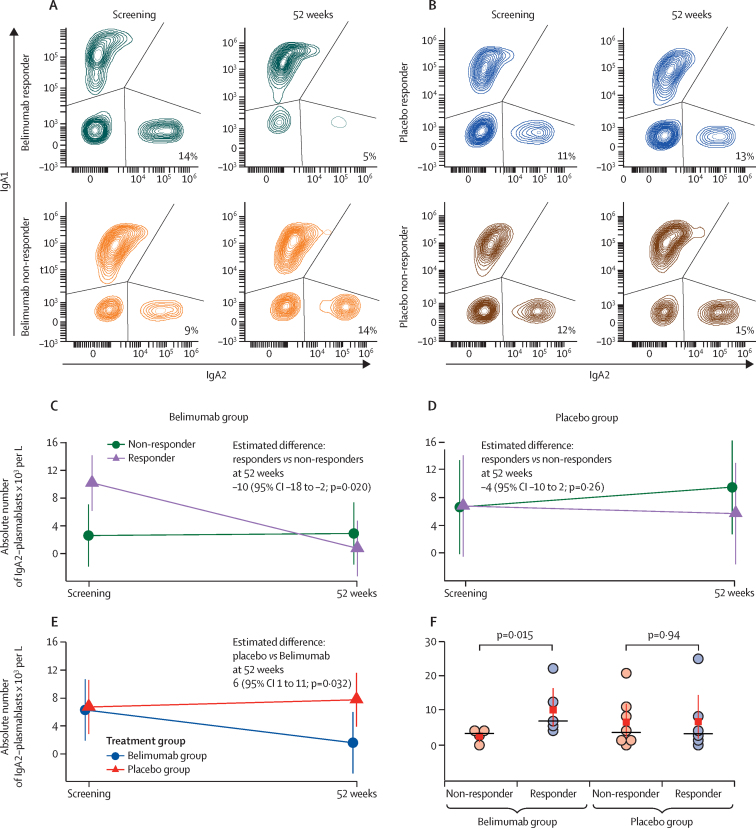
Association between numbers of IgA2-producing plasmablasts in peripheral blood at baseline and major clinical response (A, B) Representative flow cytometry plots of IgA1-secreting and IgA2-secreting plasmablasts (gated as CD19^+^CD27^hi^CD38^hi^) at screening and at 52 weeks, stratified by belimumab (after rituximab) responders and non-responders (A) and placebo (after rituximab) responders and non-responders (B). Cumulative data of absolute number of IgA2-secreting plasmablasts at screening and 52 weeks show for responders (n=5) and non-responders (n=5) in the belimumab group (C), responders (n=6) and non-responders (n=7) in the placebo group (D), and belimumab versus placebo (E), both after rituximab. A linear regression analysis of covariance model was fitted and adjusted for baseline values, age, sex, concomitant mycophenolate (yes or no), and prednisolone dose at the two timepoints to calculate expected difference at 52 weeks. Estimated means with 95% CIs are shown, with p value at weeks 52. (F) Comparison of absolute number of IgA2-secreting plasmablasts at screening categorised according to clinical response at 52 weeks to belimumab and placebo. The boxes and bars indicate mean with 95% CI and the horizontal lines indicate the median value. p values are shown above by non-parametric pairwise comparison, calculated using Dunn's multiple comparison test with Bonferroni's adjustment

We next explored the cellular basis for the changes in serum IgA2 anti-DNA antibodies through analysis of the dynamics of IgA2-secreting peripheral blood plasmablasts divided according to response at 52 weeks (for gating strategy see appendix p 22). We found a significant reduction in the number of IgA2-secreting peripheral blood plasmablasts at 52 weeks in patients treated with belimumab who had a major clinical response compared with non-responding patients (figure 4A–C). By contrast, we found no change in the number of IgA2-secreting plasmablasts at 52 weeks in patients treated with placebo after rituximab irrespective of response (figure 4D). The absolute number of peripheral blood IgA2-secreting plasmablasts reduced in the belimumab group at 52 weeks compared with the placebo group (figure 4E). The absolute number of IgA2-secreting plasmablasts was higher at screening in patients treated with belimumab, but not those given placebo, who had a major clinical response at 52 weeks (figure 4F). By contrast with IgA2-secreting plasmablasts, the number of total plasmablasts did not decrease between baseline and week 52 with either therapy; although, in the placebo group, non-response was associated with an increased number of total plasmablasts from baseline (appendix p 22). The baseline number of total plasmablasts was not associated with clinical response at 52 weeks in either group (appendix p 22).

We analysed circulating T follicular helper cells as key drivers of plasmablast differentiation. T follicular helper cells were defined as CD4^+^CXCR5^+^ICOS^+^PD1^+^ using flow cytometry (for gating strategy see appendix p 23). We found a significant reduction from baseline to 52 weeks in the absolute number of T follicular helper cells in patients in the belimumab group compared with those in the placebo group (estimated difference of 12 × 10^9^ cells per L [95% CI 1 to 23]; p=0·039; appendix p 24). The number of T follicular helper cells was significantly reduced in patients in the belimumab group who had a major clinical response compared with non-responders (estimated difference of –9 × 10^9^ cells per L [95% CI –18 to –1]; p=0·044), whereas we found no difference between responders and non-responders in the placebo group (estimated difference of –8 [–30 to 13]; p=0·42; appendix p 24). The number of T follicular helper cells was higher in responders than in non-responders at baseline in the belimumab group, but there was no difference at baseline between responders and non-responders in the placebo group (appendix p 24).

We have previously shown that patients treated with belimumab after rituximab were more likely to have a renal response or no new renal flare (according to BILAG–2004 A or B in the renal domain), or both, than were patients given placebo after rituximab.^5^ By contrast, in the current analysis, the favourable effect of belimumab versus placebo was not observed for mucocutaneous or musculoskeletal disease (appendix p 25). We observed an improvement in renal disease with belimumab, which was most clearly observed between week 40 and 52, but not for mucocutaneous or musculoskeletal disease activity (appendix p 25). These differences prompted us to use clinical and laboratory variables based on disease status, concomitant treatment, biochemical and immunological profile, serum antibodies, serum total immunoglobulins, serum cytokines, and IFN score and BAFF RNA expression (listed in the appendix [p26]) to identify biomarkers associated with specific organ involvement at screening.

**Figure 5:**
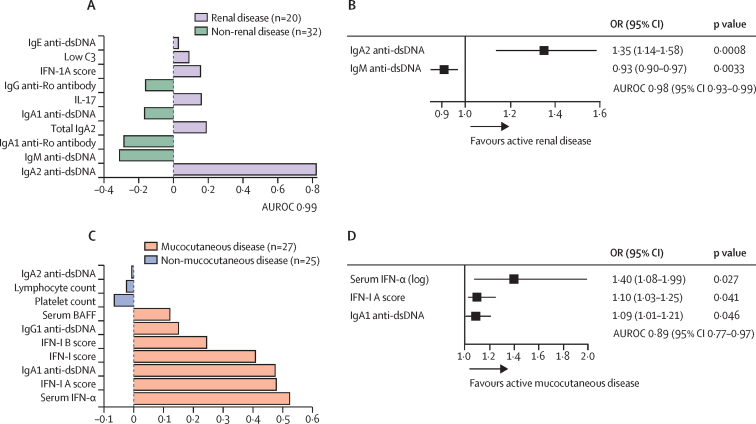
Top variables predicting active renal and mucocutaneous disease at screening (A, C) Sparse partial least squares discriminant analysis, with factor-loading weights in component 1 are shown for the top 10 ranked parameters that predicted active renal disease (A) and mucocutaneous disease (C) at baseline (ie, at screening). (B, D) OR with 95% CIs of multiple logistic regression to construct the final model to predict active renal disease at screening (B) or active mucocutaneous disease at screening (D), where variables were selected by the random forest classification algorithm. Unit changes for the continuous variables used in the logistic regression are shown the appendix (p 6). AUROC=area under the receiver operator curve. dsDNA=double-strand DNA. OR=odds ratio.

The serum IgA2 anti-dsDNA antibody concentration was the most important variable associated with active renal disease at baseline (figure 5A). The OR of active renal disease was 1·17 (95% CI 1·08–1·29; p<0·0001) for each AU increase in serum IgA2 anti-dsDNA antibody concentrations (appendix p 27). Complete-case analysis for parameters with missing values are shown in the appendix (p 27). By RRF, the importance of serum IgA2 anti-dsDNA antibody concentration was greater than all the other variables analysed (appendix p 27). Serum IgA2 and IgM anti-dsDNA antibody concentrations were selected using the random forest algorithm and fitted into the final model (AUROC 0·98 [95% CI 0·93–0·99; figure 5B). In multivariate analysis, each 1 AU increase in serum IgA2 anti-dsDNA antibody concentration increased the risk of active renal disease, whereas a 1 AU increase in serum IgM anti-dsDNA antibody concentration reduced the risk (figure 5B). Serum from an independent cross-sectional cohort were used to validate the association between serum IgA2 and IgM anti-dsDNA antibody concentration and renal involvement. Serum IgA2 anti-dsDNA antibody concentrations were significantly higher in patients with active renal disease than in those without active renal disease in both the BEAT-LUPUS and the independent organ involvement validation cohort, whereas serum IgM anti-dsDNA antibody concentrations were lower in those with active renal disease in both patient cohorts (appendix p 28). We stained renal biopsy samples for IgA1 and IgA2 from five patients with lupus nephritis selected on the basis of glomerular IgA deposits. IgA1 deposits were detected in five (100%) of five patients and IgA2 deposits were detected four (80%) of five patients (appendix p 29).

We next sought to identify a molecular signature that was associated with active mucocutaneous disease. Serum IFN-α concentrations, and type 1 interferon (IFN-1) induced expression signatures, specifically total IFN-score and type 1A score, and IgA1 anti-dsDNA antibody concentrations were the dominating variables predictive of active mucocutaneous disease (figure 5C) and their ORs were significant both from imputed and complete case analysis (appendix p 30). By RRF, the importance of these variables was greater than other variables (appendix p 30). Serum IFN-α, IFN-1A expression signature, and IgA1 anti-dsDNA antibody were selected with the random-forest algorithm and fitted in the final model (figure 5D). Serum IFN-α and IFN-1A score were predictive of mucocutaneous disease, and each 1 AU increase in serum IgA1 anti-dsDNA antibody concentration increased the risk of mucocutaneous disease (figure 5D; multivariate model with complete-case analysis is in the appendix [p 30]). To validate the association between serum IgA1 anti-dsDNA antibody concentration and mucocutaneous involvement, we analysed serum levels of IgA1 anti-dsDNA antibody in the organ involvement validation cohort. Serum IgA1 anti-dsDNA antibody concentrations were significantly higher in patients with active mucocutaneous disease (appendix p 28). Belimumab did not significantly suppress serum IgA1 anti-dsDNA antibody concentrations, IFN expression signatures, or serum concentrations compared with placebo (appendix pp 18, 31).

We correlated numerical BILAG-2004 scores,^14^ designed to assess disease activity in different organs and systems, using screening variables as listed in the appendix (p 26). Serum IgA2 anti-dsDNA antibody concentrations had the strongest positive correlation with numerical BILAG-2004 score in the renal domain at screening (Spearman's correlation coefficient [*r*] 0·68 [95% CI 0·48–0·79]; p=0·0007; appendix p 32). The mucocutaneous numerical BILAG-2004 score was positively correlated with serum IFN-α (*r* 0·43 [0·36–0·64]; p=0·0082), IFN-1 total score (0·40 [0·33–0·62]; p=0·0094, and IgA1 anti-dsDNA antibody concentrations (0·39 [0·31–0·52]; p=0·017).

In view of the substantial proportion of patients who did not have a major clinical response, irrespective of their treatment, we sought to identify the key variables that were associated with not reaching a major clinical response at 52 weeks in the whole trial cohort. Serum IL-6 concentration at baseline emerged as the only variable significantly associated with an unfavourable response (OR for major clinical response 0·38 [95% CI 0·16–0·93]; p=0·033; appendix p 33). IL-6 (log-transformed) was identified as a negative predictor when the placebo group was analysed separately (figure 1F), but did not reach significance for belimumab (figure 1B, C; complete-case analysis is shown in the appendix [p 34]). Serum IL-6 concentrations remained unchanged from screening to 52 weeks in both groups of the trial, regardless of response (appendix p 35). We found a greater reduction in serum IL-12 concentration in the belimumab group at 52 weeks than in the placebo group (estimated difference 0·48 [95% CI 0·14–0·82]; p=0·0063); however, these changes were not associated with clinical response (appendix p 35).

## Discussion

SLE is one of the most diverse autoimmune diseases, with numerous different presentations and variable organ involvement in individual patients. This heterogeneity is reflected by divergent responses to treatment, including to targeted therapies. We used a machine learning approach, with an array of clinical and laboratory data from patients in the BEAT-LUPUS trial, and identified serum IgA2 anti-dsDNA antibodies to be the key positive baseline predictor of response to belimumab after rituximab in patients with SLE. We also observed a substantial decrease in their concentration from baseline to 52 weeks only in patients who had a major clinical response. IgA2 outperformed other anti-dsDNA antibody isotypes, including the routinely measured IgG anti-dsDNA antibody, which had no predictive value, in predicting response to this combination of B-cell-targeted therapies. The substantially greater difference (48%) in treatment outcome between the belimumab and placebo group in patients with high baseline IgA2 anti-dsDNA antibody concentrations than when analysing without a biomarker (13%) shows the potential of a simple assay measuring IgA2 anti-dsDNA antibody concentration and how it could be applied in clinical practice to guide patient selection for rituximab followed by belimumab combination therapy. However, these findings need to be confirmed in other studies.

A wealth of evidence indicates that the IgG anti-dsDNA antibody isotype is associated with active SLE, particularly renal disease, and is used as a biomarker to guide belimumab therapy.^2^, ^15^ By contrast, there is a paucity of data supporting the association between IgA anti-dsDNA antibodies and renal disease in SLE,^16^, ^17^ and none to our knowledge that have assessed IgA1 and IgA2 anti-dsDNA subclasses separately, which could explain discrepancies in this small pool of literature. The association between IgA and SLE has been noted using a variety of approaches. Circulating IgA plasmablasts are found in individuals with SLE, some of which secrete anti-dsDNA antibodies, and the plasmablasts can be detected in inflamed kidneys.^18^, ^19^ BAFF can promote class switching to IgA2,^20^ including acting synergistically with APRIL,^21^ and overexpression of BAFF leads to increased levels of commensal flora-specific serum IgA and the development of nephropathy.^22^ The differential role of IgA1 and IgA2 is beginning to be investigated in other autoimmune diseases (but not in mice because they only have one IgA subclass). IgA2 can induce neutrophil extracellular trap (NET) formation together with cytokine production by neutrophils and macrophages in individuals with rheumatoid arthritis.^23^ Indeed, the combination of belimumab after rituximab has been shown to reduce excessive NET formation in patients with SLE.^24^ IgA antibodies are important for immune defence at mucosal surfaces such as the gut, and could have a vital role in the association between the microbiome and the onset or worsening of SLE disease. Both IgA subclasses bind similar commensal bacteria in the small intestine, although in the colon IgA2 preferentially targets several genera compared with IgA1 in healthy individuals.^25^ IgA1 is the dominant IgA subclass in the serum,^26^ but we found that IgA1 anti-dsDNA antibody concentrations were unaffected by belimumab after rituximab therapy, which is consistent with the hypothesis that BAFF blockade after rituximab specifically targets IgA2 autoantibody formation, perhaps initially in the colon. Although we found IgA2 and IgA1 deposited in the glomeruli of some patients with lupus nephritis, the link between IgA2 and SLE could also be indirect and might reveal more about the initiation of lupus nephritis triggered by specific microbiota in the colon. Enumeration of circulating plasmablasts secreting IgA1 and IgA2 and T follicular helper cells supported our serological analyses, suggesting not only that inhibition of BAFF targets autoreactive interactions between T cells and B cells, but also that IgA2-secreting plasmablasts are present systemically, possibly having migrated from the colon. Circulating T follicular helper cells can stimulate the differentiation of B cells into plasmablasts in the context of SLE.^27^

In addition to our major finding on the importance of serum IgA2 anti-dsDNA antibodies in predicting response to belimumab after rituximab and its association with renal disease, stratification of the patients in the BEAT-LUPUS trial uncovered other important correlates of disease and response to B-cell-targeted therapy. We found an association between IFN-α, serum IgA1 anti-dsDNA antibody concentrations, and mucocutaneous disease, consistent with previous findings.^28^, ^29^ The most striking baseline variable associated with non-response in the BEAT LUPUS trial was serum IL-6 concentrations, which were unaffected during treatment with belimumab or placebo after rituximab. This finding is in contrast with a previous report in which IL-6 concentrations decreased in response to belimumab alone;^30^ although, rituximab is known to trigger release of IL-6.^31^ Notably, high serum IL-6 concentrations have been associated with non-response to rituximab in rheumatoid arthritis.^32^

This study has several limitations. BEAT-LUPUS was a small trial, although in an uncommon disease where academic-led trials are rare. These results require validation in a larger trial, ideally a trial in which patients are stratified according to serum IgA2 anti-dsDNA antibody concentrations. Flow cytometry could only be done on a subset of patients because of the varying sample processing capacity in each recruiting centre. If IgA2 anti-dsDNA antibodies are to be measured in routine clinical practice, the ELISA assay we used will need to be commercially validated in a similar manner to IgG anti-dsDNA antibody assays and the results confirmed in a larger clinical trial. IgA2 was associated with both renal disease and response to therapy, and although renal disease was not identified as a strong predictor of response, a larger trial is needed to dissect this association.

In summary, our analyses show how targeted therapy can be used to investigate mechanisms of action and reveal the immune pathogenesis of a SLE endotype that is specifically responsive to belimumab after rituximab combination therapy.

## Data sharing

Data that underlie the results presented here will be shared upon reasonable request while preserving patient anonymity. Requests should be sent to ctu.beatlupus@ucl.ac.uk and the corresponding author at m.ehrenstein@ucl.ac.uk. All data will be made available for a period of 3 years after publication of this Article. A proposal with a detailed description of study objectives and statistical analysis plan will be requested. After approval of a proposal, data will be shared through a secure online platform after signing a data access agreement. The codes that were used to generate the machine learning algorithms can be found online.

## Declaration of interests

MS and MRE are named on a patent pending (IgA2 anti-dsDNA antibodies as a biomarker in SLE, the patent is to University College London). MRE has received grant support from GSK, VersusArthritis, National Institute of Health and Care Research (NIHR), UK Medical Research Council (MRC), and Lupus UK, on behalf of his coauthors, which funded the research. MRE and CG have been members of the speakers’ bureau for GSK and have received consultancy fees for attending GSK advisory boards. DAI has received consultancy fees from AstraZeneca, Eli Lilly, Merck Serono, Servier. CG also reports personal fees for honoraria from consultancy work from the US Centers for Disease Control and Prevention, AbbVie, Amgen, AstraZeneca, EMD Serono, MGP, Sanofi, and UCB; personal fees for a speakers’ bureau from UCB; and an educational grant from UCB to Sandwell and West Birmingham Hospitals NHS Trust that supported her research work. All other authors declare no competing interests.
