# Supplementary material for: Identification of biomarkers to stratify response to B-cell-targeted therapies in systemic lupus erythematosus: an exploratory analysis of a randomised controlled trial
Source: Lancet Rheumatol. 2022 Nov 28;5(1):e24–35. doi: 10.1016/S2665-9913(22)00332-0 (PMC9894756; doi:10.1016/S2665-9913(22)00332-0)
Supplement: Supplementary appendix [file mmc1.pdf]

THE LANCET
Rheumatology

Supplementary appendix

This appendix formed part of the original submission and has been peer reviewed. We post it as supplied by the authors.

Supplement to: Shipa M, Santos LR, Nguyen DX, et al Identification of biomarkers to stratify response to B-cell-targeted therapies in systemic lupus erythematosus: an exploratory analysis of a randomised controlled trial. *Lancet Rheumatol* 2022; published online Nov 28. https://doi.org/10.1016/S2665-9913(22)00332-0.

Identification of biomarkers to stratify response to B cell targeted therapies in a randomized trial for systemic lupus erythematosus

Authors: Muhammad Shipa†[1], MBBS; Liliana Ribeiro Santos†[1], MD; Dao X Nguyen[1], MSc; Andrew Embleton-Thirsk[2], PhD; Mariea Parvaz[1], MSc; Lauren L Heptinstall[3], Ruth J Pepper[3], PhD; Prof. David A Isenberg[1], MD; Prof. Caroline Gordon[4], MD; and Prof. Michael R Ehrenstein, PhD[1*].

SUPPLEMENTARY MATERIALS

SUPPLEMENTARY METHODS

ELISA

A commercially available ELISA kit was used to analyse IgG, (Abnova, Taiwan, cat # KA 1100), IgM(Abnova, Taiwan, cat # KA 1099), IgA anti-dsDNA antibodies (Abnova, Taiwan, cat # KA 1098), IgG ENA (extractable nuclear antigen)-4 antibodies combi kit (2B scientific, UK, cat # KA1103), and serum BAFF (R&D Systems, cat # DY124-05)

For the in-house subclass ELISAs, 96-well maxisorb plates were precoated with 100 μl/well protamine sulphate (for anti-dsDNA ELISA). After washing, plates were coated overnight with dsDNA from calf thymus (Sigma, St Louis, MO, USA) or ENA [Ro/SS-A-antigen (2B scientific LTD, cat # A07300B-1000), La/SS-B-antigen (2B scientific LTD, cat # MD-27-0018P), Sm-antigen (2B scientific LTD, cat # GWB-D554DD), RNP/Ribonucleoprotein-antigen (2B scientific LTD, cat # 614-0159)] and blocked with BSA. Serum samples were added at a 1:100 dilution with the same positive and negative control sera included in all plates (separate positive control for anti-dsDNA and ENA ELISAs). After washing, bound antibody subclasses were detected using HRP-conjugated antihuman monoclonal IgG1 subclass (mouse, ThermoFisher, clone #HP6070, cat # MH1715), IgG2 subclass (mouse, ThermoFisher, clone #HP6014, cat # MH1722), IgG3 subclass (mouse, southernBiotech, clone #HP6050, cat # 9210-05), IgG4 subclass (mouse, ThermoFisher, clone #HP6025, cat # MA1-34437), IgA1 subclass (mouse, southernBiotech, clone #B3506B4, cat # 9130-05), IgA2 subclass (mouse, southernBiotech, clone #A9604D2, cat # 9140-05), and IgE (mouse, ThermoFisher, clone #24A, cat # SA5-10305). The IgA2 and IgA1 anti-dsDNA antibody ELISA results are reported in arbitrary units, where the mean absorbance (OD in 450 nm) of the same positive control sample, placed in each plate, was serially diluted to produce the standard curve by 4-parameter-fit to obtain the arbitrary unit of each sample. Total serum IgG1 (Human IgG1 ELISA kit, Stratech, cat #ORB564278-BOR), IgG2 (Human IgG2 ELISA kit, Stratech, cat #ORB564279-BOR), IgG3 (Human IgG3 ELISA kit, Stratech, cat #ORB564280-BOR), IgA1 (Human IgA1 ELISA kit, Stratech, cat #ORB564273-BOR), and IgA2 (Human IgA1 ELISA kit, Stratech, cat #ORB564274-BOR) were determined by commercially available ELISA kits. Serum BAFF was measured with a commercially available assay (Human BAFF/BLyS/TNFSF13B DuoSet ELISA, R&D Systems, cat # DY124-05).

Interferon signature and BAFF RNA expression

Whole blood samples were collected in Tempus RNA tubes and stored at -80°C. After thawing, RNA was extracted using Tempus RNA isolation kit. The isolated RNA was reverse transcribed to cDNA and quantitative real-time PCR was performed using an ABI Prism 7900HT sequence detection system. Expression levels of target genes were expressed relative to GAPDH. The median fold changes of seven interferon stimulated genes (ISG) (*ISG15, IFI44, RASD2, STAT1*, *SERPING1*, *BST2 and SP100*) compared to the median of 20 healthy controls were used to create a total interferon score ($\sum(RE_{subject} - RE_{hc})/SD_{hc}$, RE=relative expression)(1, 2). Interferon A and B scores were based on expression of ISG15, IFI44, RASD2 genes and STAT1, SERPING1, BST2 and SP100 genes respectively (adapted from(3)). BAFF expression was measured by real-time PCR.

Flow cytometry

Peripheral blood mononuclear cells (PBMC) were isolated by density gradient centrifugation over Ficoll (Merk) from patients recruited to the trial (where it was logistically feasible to send PBMC to a central laboratory) and cryopreserved in liquid nitrogen as previously described (4). Cells were thawed and stained with Live/ Dead Blue (ThermoFisher Scientific) before staining for CD4 (CD4 - AF488, BD Biosciences), CD3 (CD3 – BUV395, BD Biosciences), CXCR5 (CXCR5 - AF647, Biolegend), ICOS (ICOS - PE/Dazzle594, Biolegend) and PD-1 (PD-1 - BV711, Biosciences) surface expression. To analyse IgA1 and IgA2 secreting plasmablasts, PBMC were stained with CD19 (CD19 BUV395, BD Biosciences), CD27 (CD27 V450, BD Biosciences) and CD38 (CD38 BUV737, BD Biosciences), together with intracellular anti-IgA1 (IgA1 FITC, Cambridge Bioscience) and anti-IgA2 (IgA2 AF647, Cambridge Bioscience).

Immunohistological staining of renal biopsies

Paraffin-embedded specimens of formalin-fixed renal biopsy tissue were processed for immunohistochemical staining for IgA1 (abcam, clone - B3506B4; ab128791) and IgA2 (abcam, clone - RM125; ab193169) using mouse and rabbit monoclonal antibodies respectively, against human IgA1 and IgA2. The optimal time and temperature for incubation were predetermined. Renal sections cut at 3 µm were treated at 38°C firstly with Proteinase XXIV solution for 45 minutes for antigen retrieval, the solution was prepared by dissolving 1 tablet of Phosphate Buffer Saline (PBS) (Oxoid; BR0014G) in 100 mL DI water and adding 0.05 mg of Proteinase XXIV (Sigma; P8038-1G) at pH 7.15-7.25. Peroxidase (Leica BOND Polymer Refine Detection Kit; DS9800) was applied for 5 minutes to quench endogenous peroxidase activity, in a humidity chamber at room temperature, followed by the protein block (Leica Novocastra; RE7102) for 5 minutes to block non-specific staining. Monoclonal IgA1 and IgA2 antibodies were diluted 1:100 with BOND Primary Antibody Diluent (Leica; AR9352), for incubation at room temperature for 30 minutes. Post primary (Leica BOND Polymer Refine Detection Kit; DS9800) was applied for 20 minutes, followed by polymer (Leica BOND Polymer Refine Detection Kit; DS9800) for 20 minutes to conjugate with the DAB solution. BOND Wash Buffer (Leica; AR9590) was used to rinse slides in between all preceding steps. Sections were developed in freshly prepared DAB solution 1:24 after 5 minutes, by combining DAB Part 1 and DAB Part B (Leica BOND Polymer Refine Detection Kit; DS9800), rinsed in DI water and

counterstained with haematoxylin for 5 minutes. Stained slides were dehydrated and examined microscopically. The extensity and intensity of staining was evaluated at up to ×60 magnification and scored semi-quantitatively: 0, no staining; 1, weak/segmental staining; 2, moderate staining, 3, strong staining.

Supplementary Statistical methods

Prediction model

To construct an outcome-prediction and active organ-involvement model with the available variables (Supplementary table 1) we first aimed to select influential variables. To analyse this high dimensional dataset we applied machine learning algorithms. Growing numbers of studies are now using machine learning approaches for feature selection with high dimensional data and are building prediction models with the selected variables(5), even in rare diseases with small sample sizes similar to this study (6-8). We tested several machine learning algorithms with bootstrap and cross-validation – ensembles, least regression, support vectors, and naïve bayes(6). Based on the normalised Matthews correlation coefficient (nMCC) (9) we chose the two best performing machine learning algorithms, Sparse Partial Least Squares Discriminant Analysis (sPLS-DA) (10), and Regularised Random Forest (RRF) (11). To confirm the findings from the machine learning algorithms, we further applied conventional statistical analyses: logistic regression and direct comparison by Mann-Whitney U test.

Firstly, we applied sPLS-DA using mixOmics (12) package in R. 10-fold cross validation was applied to prevent model overfitting, and the number of components and variables were selected by model optimisation. Validation of the model data was randomly partitioned into 10 groups, 9 groups were used for training the model and the remaining group was tested for validation. This process was repeated for all 10 groups until each observation is used solely for validation. Forest plots (ggplot2 package in R software (13)) were used to show odds ratio (OR) with 95% confidence interval (95% CI) by univariable logistic regression of the selected variables by sPLS-DA. Where applicable (values were imputed due to missingness) and the complete case analysis results of the variables (without missing value) were presented.

To verify the findings, RRF (14) package in R was applied with maximally selected rank statistics where decision-trees were built using a bootstrap dataset consisting of randomly selected samples from the original dataset with 10,001 decision-trees for stabilisation, and allowing the same sample to be selected more than once to construct the model. Through model optimisation, the number of parameters to be randomly selected for each was defined. 10-fold cross validation for RRF was applied with the caret package (15). Samples that were not included in the bootstrap dataset were termed the Out-of-Bag dataset and were used to validate the model performance (60% of samples were randomly assigned to bootstrap and 40% as out-of-bag dataset). Mean decrease in Gini of the top 10 variables was reported, where a higher score indicates a higher importance of the variable to predict the outcome. Finally, Boruta variable selection (16) [using Boruta package (17) for R] approach of random forest classification algorithm was applied to select the important variables to predict outcome/organ-involvement. The chosen variables were then fitted into a conventional multiple logistic regression model and cross verified with a direct comparison between 2-groups using either t-test (parametric) or Mann-Whitney U test.

The OR (odds ratio) with 95% CI using multiple logistic regression approach was then presented along with area under the curve (AUC) of the receiver operator characteristic (AUROC) and 95% confidence intervals (CI) of this final model (by using cutpointr(18) package for R with 1,000 stratified bootstrap replicates). Complete case analysis of the final model was also presented.

The optimal cutpoint was derived from AUROC analysis by using cutpointr (18) package for R with 1,000 stratified bootstrap replicates. Our aim was to define the optimal cutpoint which maximized the sensitivity and specificity of the biomarker (serum IgA2 anti-dsDNA antibody levels) to predict response with minimum sensitivity–specificity difference. We adopted the metrics maximisation method (with maximize_metric function) with sensitivity-specificity metrics (with sum_sens_spec function) (19).

Principal component analysis

Principal component analysis (PCA) was used to visualise the longitudinal variance of each patient over time by their allocated treatment, with the variables listed in Supplementary Table 1 on their scaled and centred data.

Longitudinal changes

For the longitudinal changes a linear mixed- effect model was fitted to estimate the mean change from screening to 52 weeks with fixed effect of treatment group or treatment response intercepting with trial times from randomisation to 52 weeks, random patient effect to account for clustering by patients, and adjusted for screening value (if available for more than two-time points), age, gender and concomitant mycophenolate (yes or no) and prednisolone dose at each respective time point. The model was fitted via restricted maximum likelihood (REML). We used lme4(20) package for R for the mixed model. Similar generalised mixed models were fitted for non-parametric variables guided by probability distributions (specified in the respective figure sections). For the numerical BILAG-2004 (British Isles lupus assessment group – 2004) index changes through to 52 weeks, we fitted an unadjusted generalised linear mixed- effect model, with estimated OR from randomisation to 52 weeks with fixed effect of treatment group or treatment response intercepting with trial times, and random patient effect to account for clustering by patients. We used glmer function from lme4(20) package of R for the generalised mixed model.

Biomarker correlations with organ involvement

The non-parametric Spearman's rank correlation test was used to measure association among disease activities scores (total or organ specific numerical BILAG-2004 (21) and SLEDAI-2K(Systemic lupus erythematosus disease activity index 2000) or disease activity laboratory markers (ESR, platelets, and lymphocytes), isotypes/subclasses of anti-dsDNA antibodies, and B- and T-cell related cytokines, serum interferon, interferon-based scores. Results were presented with circos plot by using circlize package for R (22). Spearman's correlation was also applied for the correlations between the different anti-dsDNA antibodies and cytokines.

Missing values

For the prediction model, missing values were imputed using multivariate imputation by chained equations (23) of the Markov chain Monte Carlo method with the missing at random assumption. Forty imputed data sets were

created, analysed and the results pooled using Rubin's rules with mice (24) package for R. For the regression model, "norm.boot" function (linear regression using bootstrap) and for random-forest "rf" function was used. Multivariate imputation was constructed for the numerical variables using the same parameters mentioned in the Supplementary Table 1. The exact number of the missing values are given in Supplementary Table 1, Supplementary Table 2, and the respective figure legends. A complete case analysis was also performed where data were imputed due to missing values.

SUPPLEMENTARY TABLES AND FIGURES:

Supplementary Table 1 | Baseline (at screening[†]) variables used in the prediction model

Number of patients with clinical data at baseline and 52 weeks = 44[¶]

Demographics	Age [$], gender[††], race (Caucasian, black, and others) [††], active smoking[††]
Disease status	Disease duration at screening [$], previous rituximab[††], SLEDAI-2K[‡] (systemic lupus erythematosus disease activity index 2000)(25)
Active organ involvement[†††]	Renal[††], mucocutaneous[††], musculoskeletal[††]
Concomitant treatment	Prednisolone[††], Prednisolone dose [‡], mycophenolate[††], azathioprine[††], methotrexate[††]
Biochemical and immunological profile	Lymphocyte[‡‡], neutrophil[‡], eosinophil[‡‡], basophil[‡‡], monocyte[‡‡], platelet[‡], CD19 count[‡‡]; c-reactive protein (CRP) [‡‡], erythrocyte sedimentation rate (ESR) [‡], complement C3 (normal or low) [††], urine protein creatinine ratio (uPCR) [‡‡]
Serum autoantibodies	IgG anti-Sm[††], IgG anti-Ro[††], IgG anti-La[††], IgG Anti-RNP[††]<br>IgA1 anti-Sm[††], IgA1 anti-Ro[††], IgA1 anti-La[††], IgA1 Anti-RNP[††]<br>IgA2 anti-Sm[††], IgA2 anti-Ro[††], IgA2 anti-La[††], IgA2 Anti-RNP[††]<br>IgG anti-dsDNA[‡], IgM anti-dsDNA[‡], IgA anti-dsDNA[‡],<br>IgE anti-dsDNA[‡‡‡], IgG1 anti-dsDNA[‡‡‡], IgG2 anti-dsDNA[‡‡‡], IgG3 anti-dsDNA[‡‡‡], IgA1 anti-dsDNA[‡‡‡], IgA2 anti-dsDNA[‡‡‡]<br>*(IgG4 anti-dsDNA antibody was also measured but the values were not above those from healthy donors)*
Serum total Immunoglobulins (Ig)	IgG[‡], IgG1[‡], IgG2[‡], IgG3[‡], IgM[‡], IgA[‡], IgA1[‡], IgA2[‡]
Serum Cytokines	BAFF[‡] (B cell activating factor) *(2)*,<br>IL (interleukin)-6[‡‡] *(4)*, IL-10[‡‡] *(4)*, IL-12[‡‡] *(4)*, IL-17[‡‡] *(4)*,<br>TNF (tumour necrosis factor)-α [‡‡] *(4)*, IFN (Interferon)-α [‡‡] *(1)*, IFN-γ [‡‡] *(4)*
Interferon score and BAFF RNA expression	BAFF[‡] *(7)*, Type I IFN score (IFN-I) [‡] *(7)*, Type I A IFN score (IFN-I A) [‡] *(7)*, Type B IFN total score (IFN-I B) [‡] *(7)*,

Blue indicates these were obtained from sites, black indicates these were analysed in the central laboratory.
[†]Screening refers to the first screening visit before rituximab, randomisation (week 0) occurred 4-8 weeks after screening.
[¶] Only patients who provided clinical data at 52 weeks, so that the response can be determined, were included.
The number of missing values at screening for each variable are provided (in italics) after the respective variable, where applicable.
[††] Categorial variable.
*[†††]**Active renal disease** defined as – BILAG-2004 (British Isles lupus assessment group – 2004) index A or B in the renal domain at screening with a 24-hour urinary protein > 500mg/day, or urine protein-creatinine ratio (uPCR) > 50mg/mmol, or urine albumin-creatinine ratio (uACR) > 50mg/mmol at baseline, or active urinary sediment with uPCR > 25mg/mmol with active urinary sediment. **Active mucocutaneous** disease defined as either A or B scores in BILAG-2004 mucocutaneous domain. **Active musculoskeletal** disease defined as either A or B scores in BILAG-2004 musculoskeletal domain.*
Unit changes for the continuous variables for the logistic regression:
[$] per 1 year change, [‡] per 1 unit change, [‡‡] log-transformed, [‡‡‡] per 1 arbitrary unit change

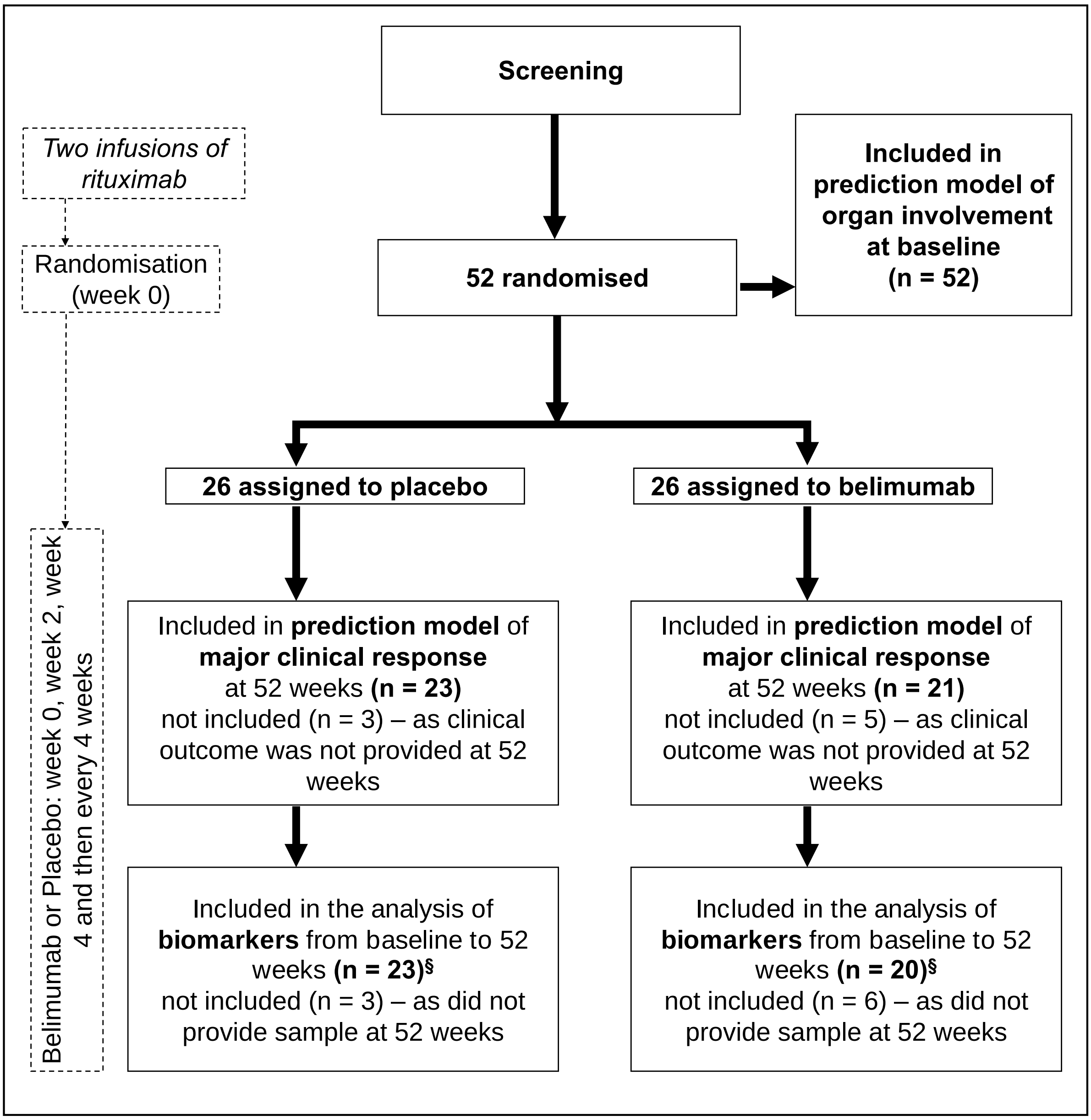

Supplementary Figure 1 | The numbers of patients included in the different analyses are shown.

[§]The numbers of patients who contributed to each set of results are presented in the relevant figure or table and depended on attendance at relevant trial visits and provision of samples.

Supplementary Table 2 | Baseline demographics and disease characteristics of the patients in the BEAT-lupus trial who provided clinical data at 52 weeks.

	Belimumab (n = 21)	Placebo (n =23)
Age, years ‡	39.5 (12.1)	42.1 (10.5)
Sex – no. of patients (%)		
Female	17 (81%)	21 (91%)
Ethnicity – no. of patients (%) §		
White	13 (62%)	16 (70%)
Black	3 (14%)	2 (9%)
South Asian	2 (10%)	1 (4%)
Chinese	1 (5%)	1 (4%)
Other	2 (9.5%)	3 (13%)
Disease duration at screening††, years		
mean (SD)	11.5 (9.1)	8.9 (7.4)
median (IQR)	10.3 (4.5-17.7)	6.2 (3.5-14.3)
Previous rituximab – no. of patients (%)	4 (19%)	7 (30%)
Previous rituximab within 2 years from screening – no. of patients (%)	2 (10%)	3 (13%)
Concomitant immunosuppressant or prednisolone at screening, no. of patients (%)		
Mycophenolate	15 (71%)	13 (57%)
Azathioprine	2 (10%)	2 (9%)
Methotrexate	3 (14%)	2 (9%)
Prednisolone	18 (86%)	21 (91%)
Receiving concomitant immunosuppressant or prednisolone	21 (100%)	23 (100%)
Concomitant hydroxychloroquine at screening, no. of patients (%)	17 (81%)	20 (87%)
Average daily prednisolone dose at screening, mg/day		
mean (SD)	12.9 (9.8)	15.1 (10.2)
median (IQR)	9 (5-18.8)	13 (8-20)
Patients taking ≥ 7.5 mg/day prednisolone, no. of patients (%) at screening	10 (48%)	16 (70%)
Patients taking ≥ 10 mg/day prednisolone at screening, no. of patients (%) at screening	9 (43%)	14 (61%)
Organ involvement (either BILAG-2004 A/B system scores) at screening, no. of patients (%)		
Constitutional	1 (5%)	2 (9%)

Cardiorespiratory		4 (19%)	6 (26%)
Mucocutaneous		10 (48%)	12 (52%)
Musculoskeletal		8 (38%)	8 (35%)
Neuro-psychiatric		0 (0%)	1 (4%)
Ophthalmic		0 (0%)	0 (0%)
Gastro-intestinal		0 (0%)	0 (0%)
Renal		10 (48%)	8 (35%)
Haematological		1 (5%)	0 (0%)
BILAG-2004 system score at screening, no. of patients (%)			
≥ 1 BILAG-2004 A		6 (29%)	12 (52%)
≥ 1 BILAG-2004 A or 2 BILAG-2004 B		13 (62%)	15 (65%)
Numerical BILAG-2004[¶] at screening			
	mean (SD)	12.4 (6.4)	14.6 (8.3)
	median (IQR)	12 (7-14)	14 (7-16)
SLEDAI-2K at screening			
	mean (SD)	10.9 (3.9)	11.3 (4.8)
	median (IQR)	12 (8-12)	12 (8-14)
Positive IgG anti-dsDNA antibody at screening, no. of patients (%)		19 (90%)	20 (87%)
IgG anti-dsDNA antibody level at screening, IU/ml			
	mean (SD)	299 (301)	227 (253)
	median (IQR)	165 (97-318)	161 (60-254)
Low complement C3 at screening, no. of patients (%)		12 (57%)	11 (48%)
Erythrocyte sedimentation rate (ESR) at screening,			
	mean (SD)	23.4 (16.5)	19.6 (15.1)
	median (IQR)	20.5 (12-28.2)	16 (10.2-22.8)
Lymphocyte count at screening, 10^9/L			
	mean (SD)	0.94 (0.53)	0.83 (0.45)
	median (IQR)	0.78 (0.58-1.28)	0.78 (0.50-0.95)
Platelet count at screening, 10^9/L			
	mean (SD)	273 (97)	273 (107)
	median (IQR)	254 (223-337)	270 (176-330)
Creatinine at screening (only renal BILAG-2004 A or B), ml/min/1.73 m^2			
	mean (SD)	60.8 (16.3)	66.8 (20.9)
	median (IQR)	56 (49-72.3)	71 (53.2-83)
eGFR at screening (only renal BILAG-2004 A or B), ml/min/1.73 m^2			
	mean (SD)	87.8 (6.6)	81.8 (9.8)
	median (IQR)	90 (90-90)	86.5 (72-90)
Urine protein/creatinine ratio at screening, mg/mmol			

	mean (SD)	191 (157)	183 (82.5)
	median (IQR)	164 (123-180)	162 (148-183)
Serum albumin at randomisation, g/L			
	mean (SD)	33.6 (5.4)	37.5 (6.8)
	median (IQR)	33.5 (32-37)	37 (33-43.5)

‡ *Mean (SD)*

§ *Reported by the patient*

†† *Screening refers to the first screening visit before rituximab*

¶ *Numerical BILAG-2004 (British Isles lupus assessment group – 2004), where BILAG-2004 A score = 12, B score = 8, C score = 1, and D/E score = 0.*

IgG = Immunoglobulin G, IQR = Interquartile range, SD = standard deviation, SLEDAI-2K = systemic lupus erythematosus disease activity index 2000

Supplementary Table 3 | Demographics and disease characteristics of the organ involvement validation cohort

	Healthy donor (n = 11)	Active renal disease (n = 6)	Active mucocutaneous disease (n = 7)
Age, years ‡	36.3 (7.2)	34.5 (6.5)	39.1 (11.2)
Female – no. of participants (%)	6 (54%)	6 (86%)	8 (89%)
Ethnicity – no. of participants (%) §			
White	7 (64%)	2 (29%)	3 (33%)
Black	0 (0%)	4 (57%)	3 (33%)
South Asian	3 (27%)	0 (0%)	2 (22%)
Chinese	1 (9%)	1 (14%)	1 (11%)
Disease duration, years			
mean (SD)	-	7.3 (5.5)	6.1 (6.3)
median (IQR)	-	6.3 (2.4-11.2)	6 (2.3-10.3)
Concomitant immunosuppressant or prednisolone, no. of patients (%)			
Mycophenolate	-	3 (43%)	2 (22%)
Azathioprine	-	3 (43%)	4 (44%)
Methotrexate	-	0 (0%)	3 (33%)
Prednisolone	-	7 (100%)	6 (67%)
Any concomitant immunosuppressant	-	6 (86%)	9 (100%)
Receiving concomitant immunosuppressant or prednisolone	-	7 (100%)	9 (100%)
Concomitant hydroxychloroquine, no. of patients (%)	-	5 (71%)	8 (89%)
Average daily prednisolone dose at screening, mg/day			
mean (SD)	-	13.2 (8.8)	10.1 (7.2)
median (IQR)	-	10.2 (4-14)	9 (5-13)
Positive anti-dsDNA antibody IgG antibody, no. of patients (%)	-	7 (100%)	9 (100%)
Low complement C3 at screening, no. of patients (%)	-	7 (100%)	8 (89%)

‡ Mean (SD)
§ Ethnicity was reported by the patient
IgG = Immunoglobulin G, IQR = Interquartile range, SD = standard deviation

Supplementary Table 4| Demography and disease characteristics of the belimumab only cohort

		Number of patients (n = 21)
Age, years ‡		32.3 (13.2)
Female – no. of participants (%)		20 (95%)
Ethnicity – no. of participants (%) §		
	White	6 (29%)
	Black	9 (43%)
	South Asian	3 (14%)
	Chinese	3 (14%)
Disease duration, years		
	mean (SD)	- 27.2
	median (IQR)	- 24.4
Concomitant immunosuppressant or prednisolone, no. of patients (%)		
	Mycophenolate	- 15 (71)
	Azathioprine	- 5 (24%)
	Methotrexate	- 1 (5%)
	Prednisolone	- 18 (86%)
	Any concomitant immunosuppressant	- 21 (100%)
	Receiving concomitant immunosuppressant or prednisolone	- 21 (100%)
Concomitant hydroxychloroquine, no. of patients (%)		- 20 (95%)
Average daily prednisolone dose at screening, mg/day		
	mean (SD)	- 14.3
	median (IQR)	- 7.5
Positive anti-dsDNA antibody IgG antibody, no. of patients (%)		- 21 (100%)
Low complement C3 at screening, no. of patients (%)		- 20 (95%)

‡ Mean (SD)
§ Ethnicity was reported by the patient
IgG = Immunoglobulin G, IQR = Interquartile range, SD = Standard deviation.

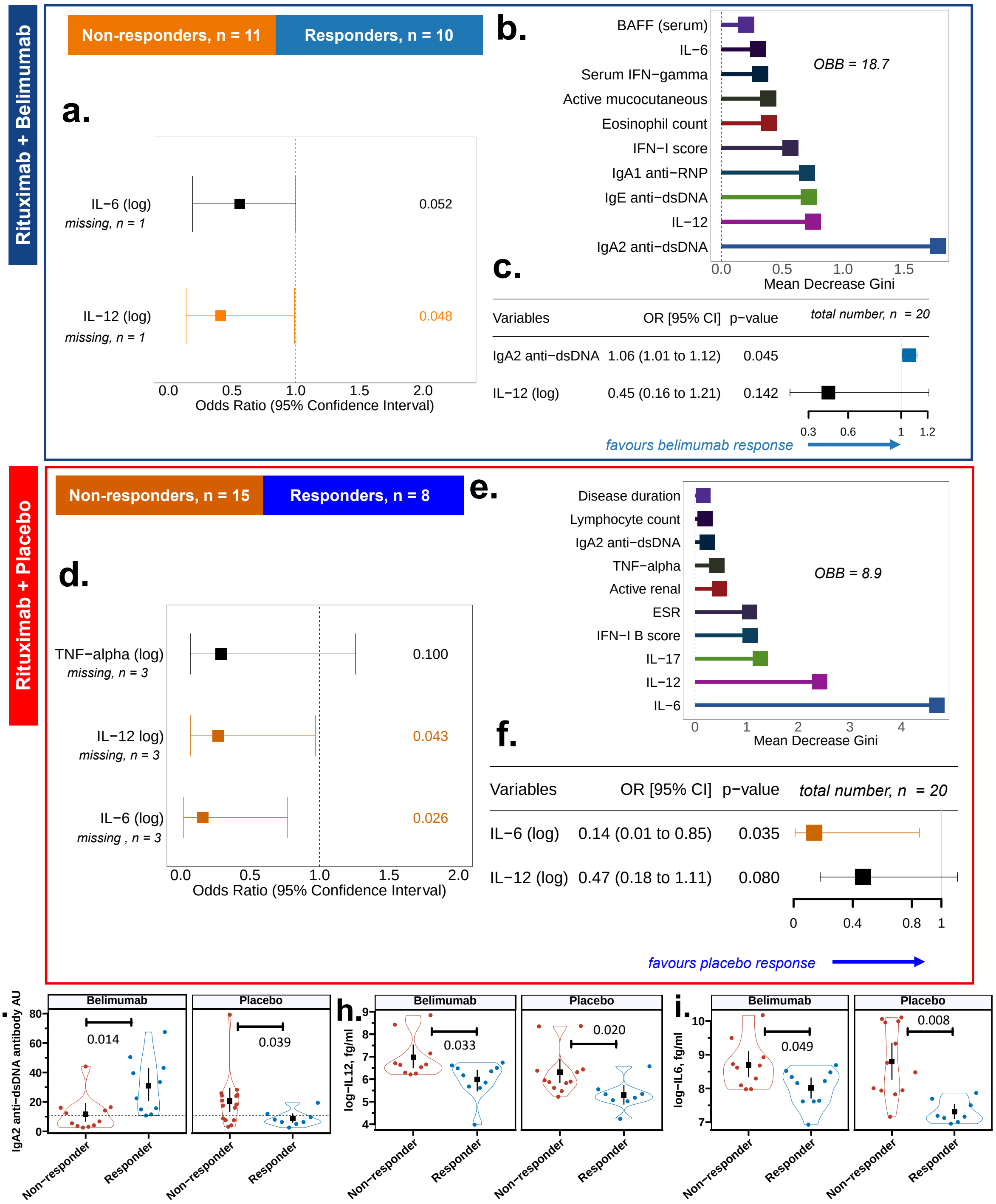

Supplementary Figure 2a-i | Supportive analysis of baseline predictors of major clinical response to belimumab following rituximab, and placebo following rituximab, at 52 weeks in the BEAT-lupus trial. Belimumab: (a) Forest plot (complete case) - odds ratios (OR) with 95% confidence interval (95% CI), by univariate logistic regression† [3 of the 5 variables, chosen by Partial Least Squares Discriminant Analysis (sPLS-DA) had missing values]. **(b)** Regularised Random Forest (RRF) - Top 10 variables by mean decrease in Gini (ranked) represents the importance of each variable to predict the belimumab response. **(c)** Multiple logistic regression† (complete case analysis) to construct the final model to predict belimumab response at 52 weeks where variables were selected by random forest classification algorithm. **Placebo: (d)** Forest plot (complete case analysis) - OR with 95% confidence interval (95% CI), by univariate logistic regression† [3 of the 5 variables,

chosen by sPLS-DA had missing values]. **(e)** Regularised Random Forest (RRF) - Top 10 variables by mean decrease in Gini (ranked) represents the importance of each variable to predict the placebo response. **(f)** Multiple logistic regression† (complete case analysis) to construct the final model to predict placebo response at 52 weeks where variables were selected by random forest classification algorithm. Comparison of **(g)** serum IgA2 anti-dsDNA antibody, **(h)** serum IL-12, **(i)** and serum IL-6 between responders and non-responders, stratified by treatment. P-values are provided tested by Mann-Whitney U test. Black boxes indicate mean with 95% CI and Black dotted line indicates upper limit of normal (3 standard deviation above the mean of healthy control samples).

† Unit changes for the continuous variables used in the logistic regression are shown in supplementary table 1.

BAFF = B-cell activating factor, ESR = Erythrocyte sedimentation rate, IFN = Interferon, IFN-I B score = Type I B interferon score, Ig = Immunoglobulin, IL = interleukin, OBB = out of bag error, RNP= Ribonucleoprotein, TNF = Tumour necrosis factor.

a: Low IgA2 anti-dsDNA

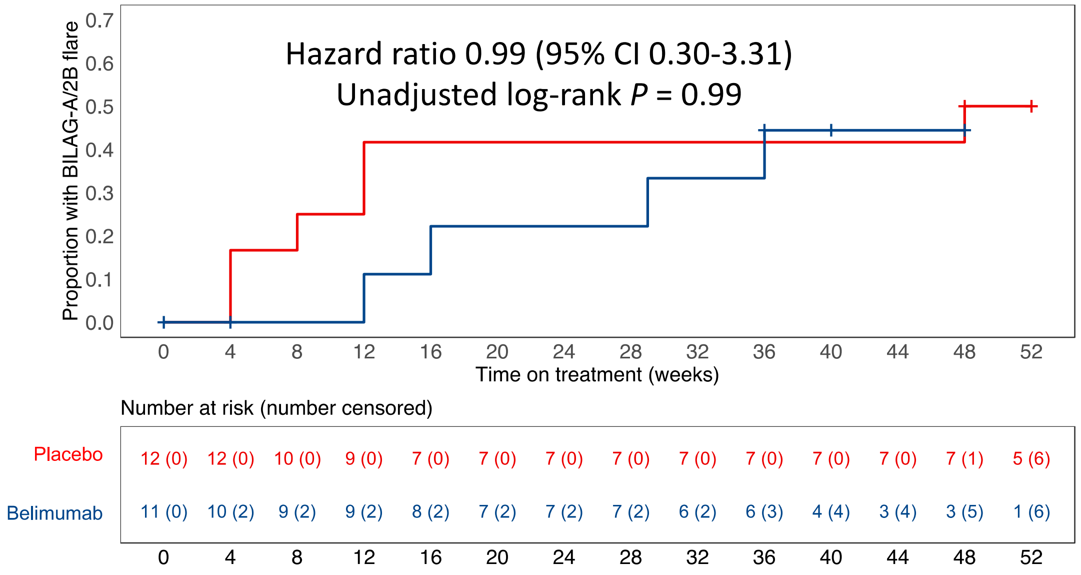

b: High IgA2 anti-dsDNA

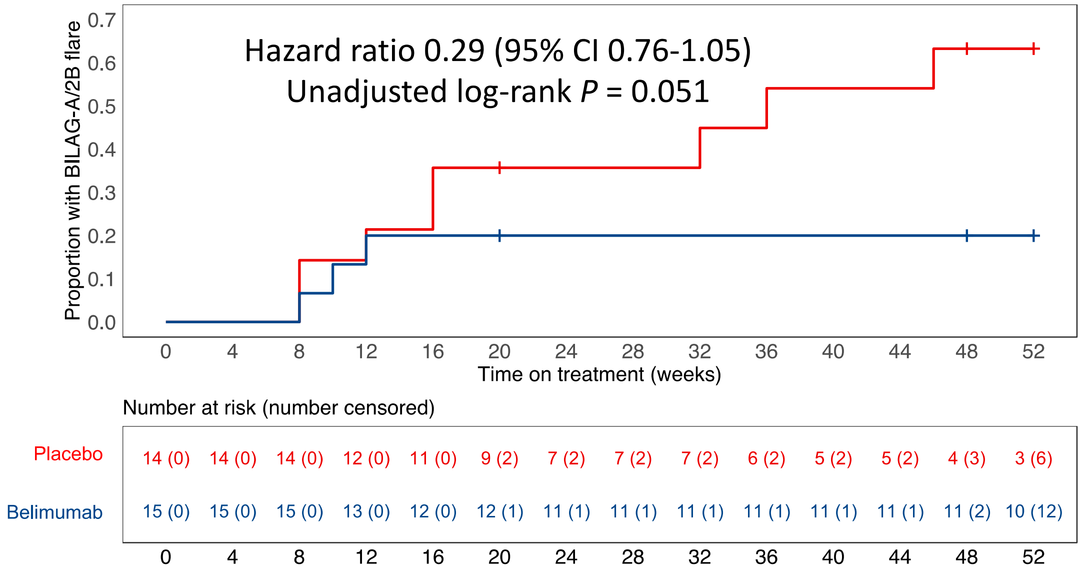

Supplementary Figure 3a-b | Kaplan-Meier curves – time to first moderate-to-severe flare (defined as either ≥1 BILAG-2004 A score or ≥2 BILAG-2004 B) stratified according to high or low serum IgA2 anti-dsDNA antibody levels. Moderate-to-Severe flares are stratified by - **(a)** low serum IgA2-antidsDNA antibody levels and **(b)** with high serum IgA2-antidsDNA antibody levels. Definition of high and low serum IgA2-antidsDNA antibody levels is based on the cutpoint derived from Figure 2a :10.7 AU/arbitrary units.

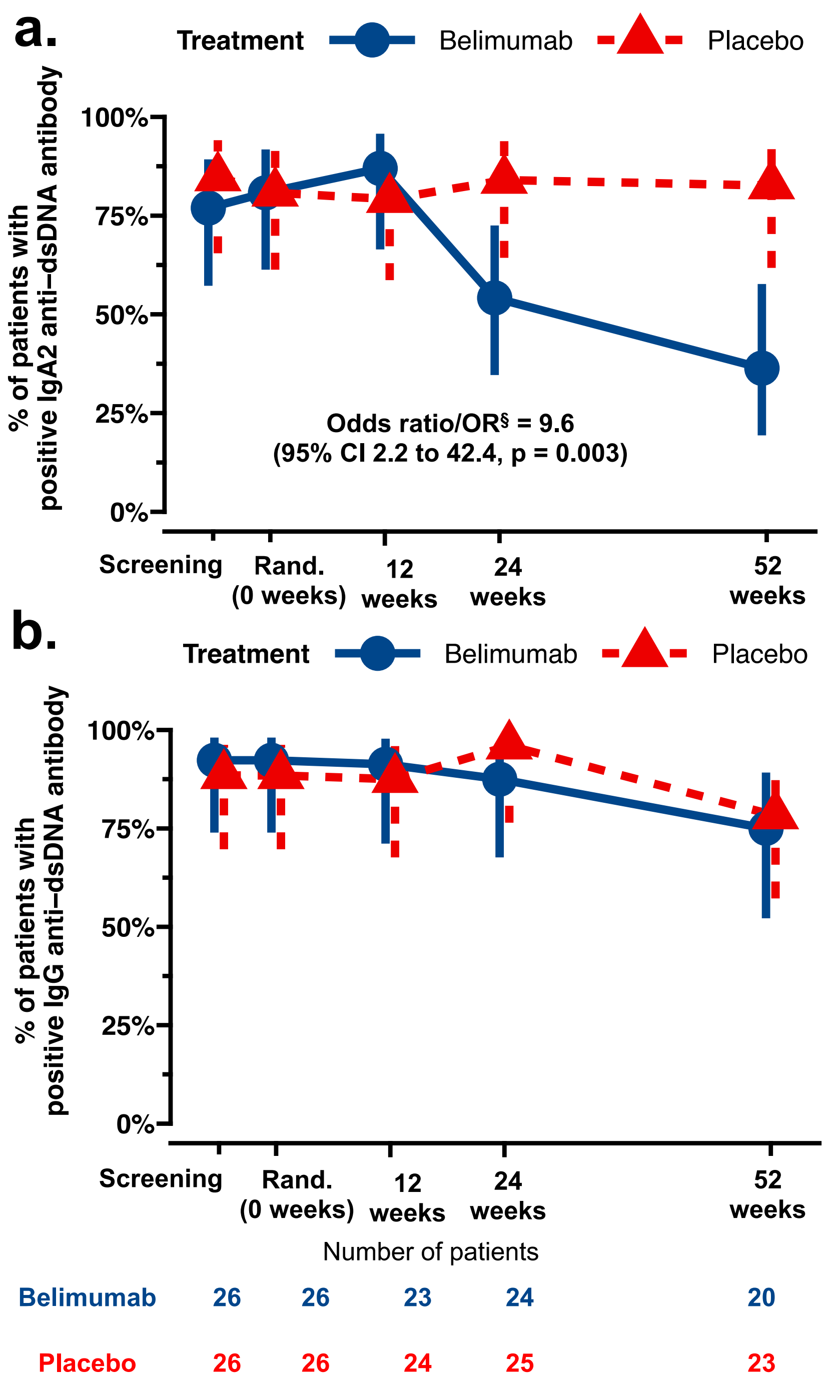

Supplementary Figure 4a-b | The percentage of patients who were positive† for IgA2 anti-dsDNA antibodies was significantly reduced at 52 weeks after belimumab (and rituximab) therapy, but the percentage of patients who were positive†† for IgG anti-dsDNA antibody remained unchanged. Comparison at 52 weeks was done by fisher-exact test and the p value is shown.

§ Odds ratio (OR) of seronegative reversion for IgA2 anti-dsDNA antibodies at 52 weeks with belimumab compared to placebo (adjusted by number of seropositive IgA2 anti-dsDNA antibody patients at screening).

†Positivity was defined as 3 standard deviation above the mean values of healthy control samples (=5.4 arbitrary units).

††Positivity was defined as per the manufacturer provided value (20 IU/ml).

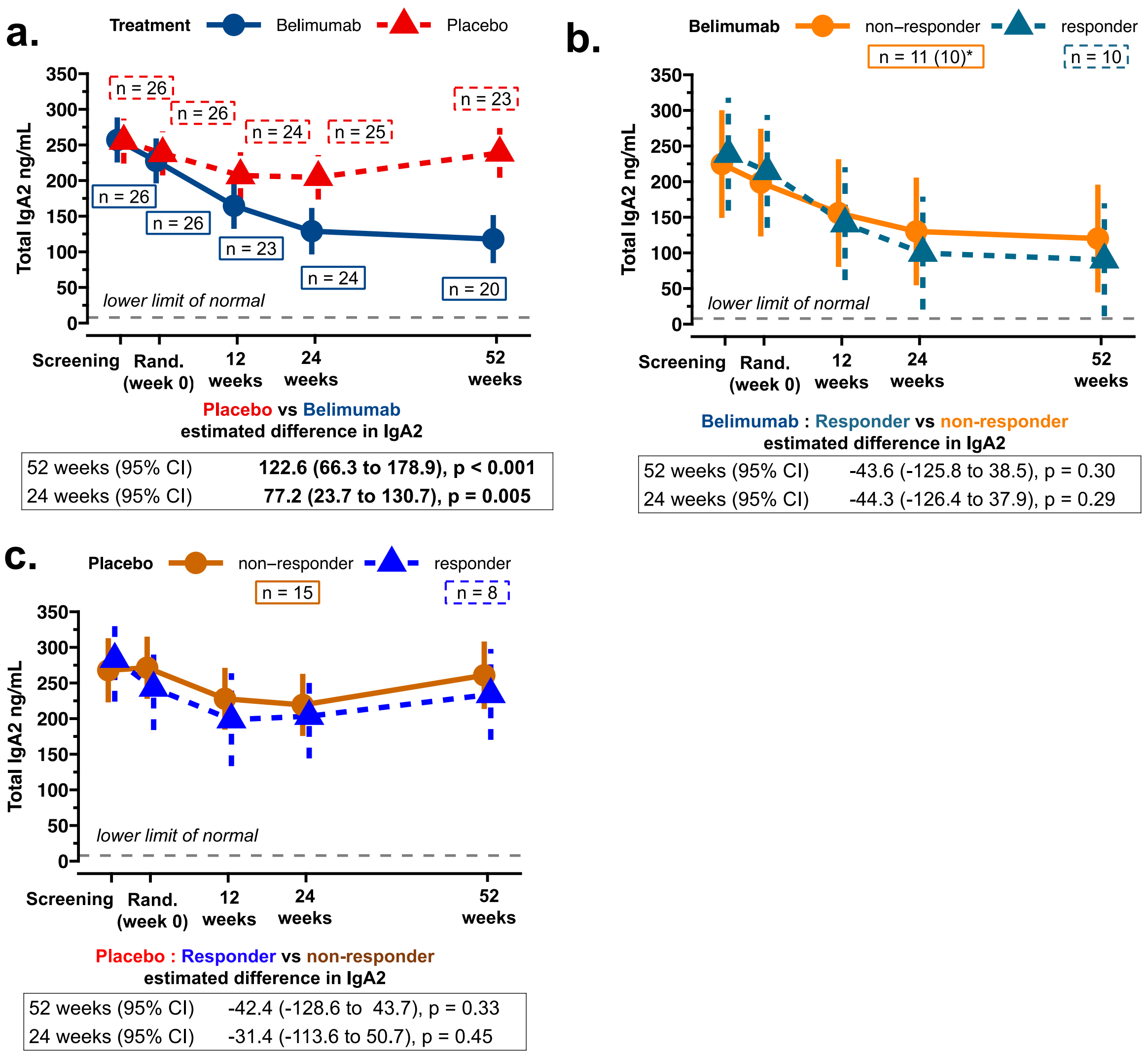

Supplementary Figure 5a-c | Serum total IgA2 (immunoglobulin A2) levels between screening and 52 weeks. Total IgA2 levels from screening to 52 weeks stratified by - **(a)** treatment either to belimumab or placebo (after rituximab), **(b)** major clinical responders and non-responders in the belimumab arm, and **(c)** responders and non-responders in the placebo arm. A longitudinal linear mixed-effect model was fitted with random patient effect to account for clustering by patients, and fixed effect of treatment group, intercepting with trial times and adjusted for screening value, age, gender, concomitant mycophenolate (yes or no), and prednisolone dose at the indicated time points to calculate expected difference at 24 and 52 weeks. Estimated mean with 95% confidence intervals and number of patients at each time points (n) are shown, and the p values at weeks 24 and 52 are provided. The black dotted line indicates the lower limit of the normal (as per the manufacturer).

* All the samples were available from screening to 24 weeks, except one sample was unavailable at 52 weeks.

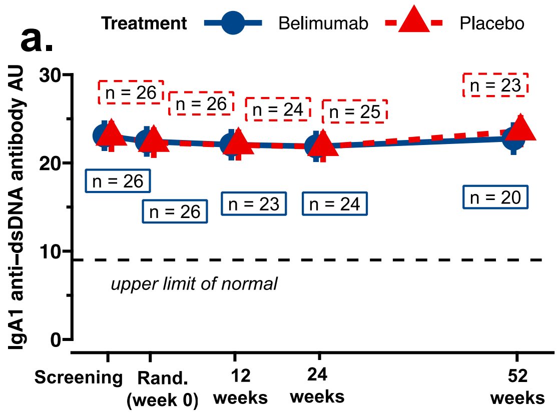

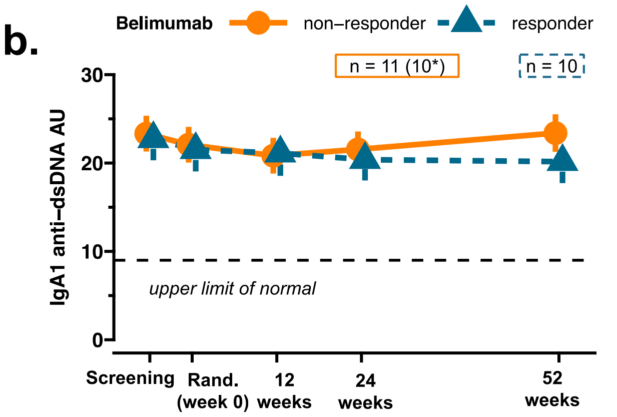

Placebo vs **Belimumab**
estimated difference in IgA1 anti-dsDNA

52 weeks (95% CI)	1 (-2 to 4), p = 0.46
24 weeks (95% CI)	0 (-2 to 2), p = 0.97

Belimumab : Responder vs **non-responder**
estimated difference in IgA1 anti-dsDNA

52 weeks (95% CI)	-3 (-7 to 1), p = 0.181
24 weeks (95% CI)	-1 (-4 to 3), p = 0.76

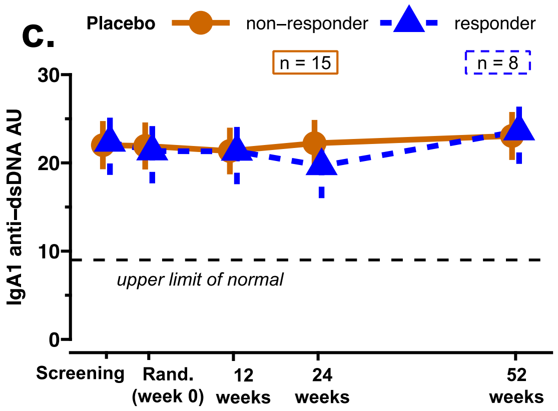

Placebo : Responder vs **non-responder**
estimated difference in IgA1 anti-dsDNA

52 weeks (95% CI)	0.2 (-3 to 4), p = 0.89
24 weeks (95% CI)	-3 (-6 to 1), p = 0.104

Supplementary Figure 6a-c | Serum IgA1 anti-dsDNA antibody levels between screening and 52 weeks. Serum IgA1 anti-dsDNA antibody levels (expressed as AU = arbitrary units) from screening to 52 weeks stratified by - **(a)** treatment with belimumab or placebo (both after rituximab), **(b)** major clinical responders and non-responders in the belimumab arm, and **(c)** responders and non-responders in the placebo arm. Longitudinal linear mixed-effect model was fitted with random patient effect to account for clustering by patients and fixed effect of treatment group intercepting with trial times and adjusted for screening value, age, gender, concomitant mycophenolate (yes or no), and prednisolone dose at indicated time points to calculate expected difference at 24 and 52 weeks. Estimated mean with 95% confidence intervals and number of patients at each time points (n) are shown, and the p values at weeks 24 and 52 are provided. The black dotted line indicates the upper limit of the normal (3 standard deviation above the mean of healthy control samples).

* All the samples were available from screening to 24 weeks, except one sample was unavailable at 52 weeks.

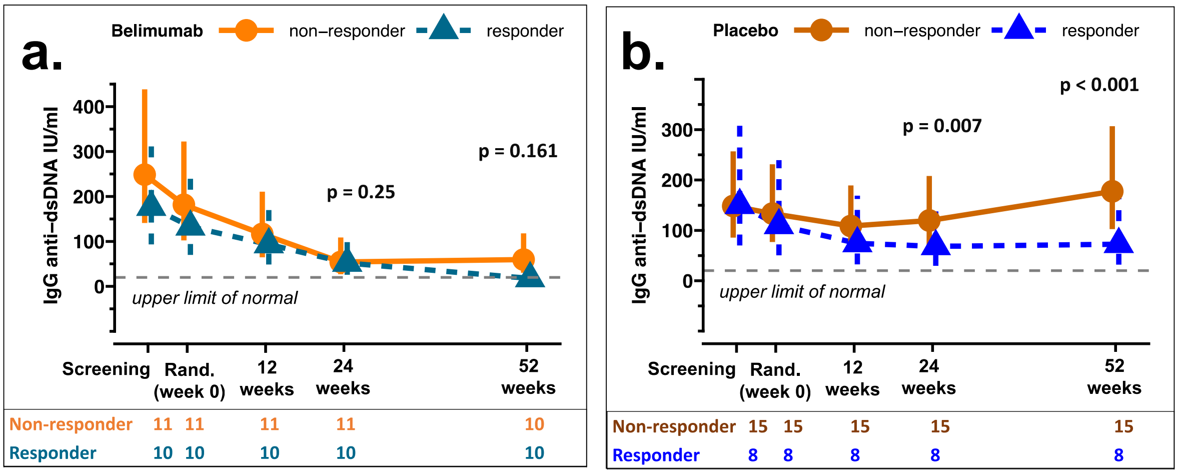

Supplementary Figure 7a-b | Serum IgG anti-dsDNA antibody levels between screening and 52 weeks. Serum IgG anti-dsDNA antibody levels from screening to 52 weeks stratified by - **(a)** major clinical responders and non-responders in the belimumab arm, and **(b)** responders and non-responders in the placebo arm. Longitudinal generalised linear mixed-effect model (log distributed) was fitted with random patient effect to account for clustering by patients and fixed effect of treatment group intercepting with trial times and adjusted for screening value, age, gender, concomitant mycophenolate (yes or no), and prednisolone dose at respective time points to calculate expected difference at 24 and 52 weeks. Estimated mean with 95% confidence intervals and number of patients at each time points (n) are shown, and the p values at weeks 24 and 52 are provided. The black dotted line indicates the upper limit of the normal (as per the manufacturer provided value).

* All the samples were available from screening to 24 weeks; one sample was unavailable at 52 weeks.

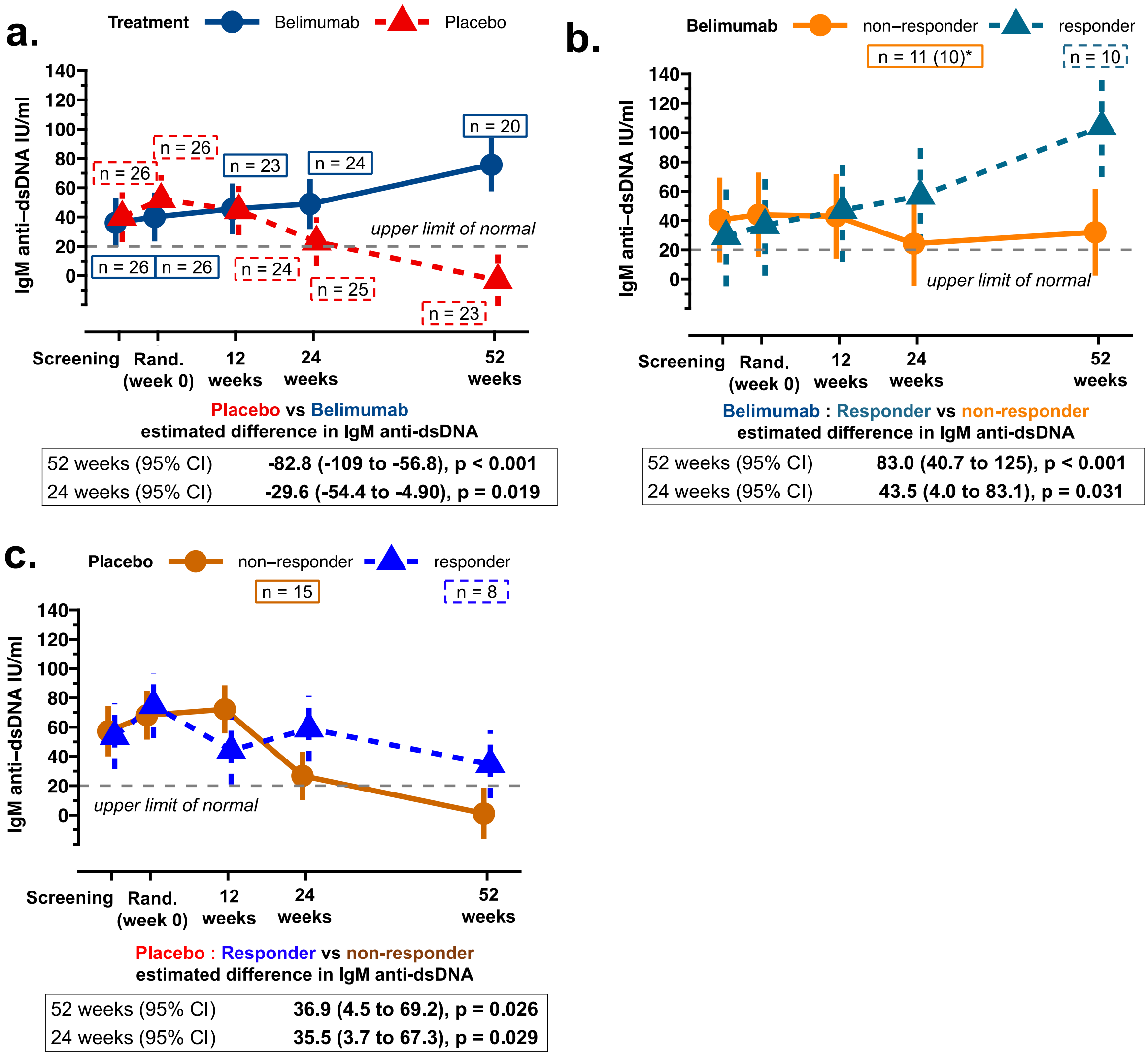

Supplementary Figure 8a-c | Serum IgM anti-dsDNA antibody levels between screening and 52 weeks. Longitudinal change of serum IgM anti-dsDNA antibodies stratified by - **(a)** treatment i.e., belimumab versus placebo (after rituximab), **(b)** treatment response in belimumab treated group, and **(c)** treatment response in placebo treated group. A longitudinal generalised linear mixed-effect model was fitted with random patient effect to account for clustering by patients and fixed effect of treatment group intercepting with trial times and adjusted for screening IgM anti-dsDNA antibody values, age, gender, concomitant mycophenolate (yes or no), and prednisolone dose at respective time points to calculate expected difference at 24 and 52 weeks in IgM anti-dsDNA. Estimated mean with 95% confidence intervals and number of patients at each time points (n) are shown; p values at weeks 24 and 52 are provided. The black dotted line indicates the upper limit of the normal (as per the manufacturer provided value).

* All the samples were available from screening to 24 weeks, except one sample was unavailable at 52 weeks.

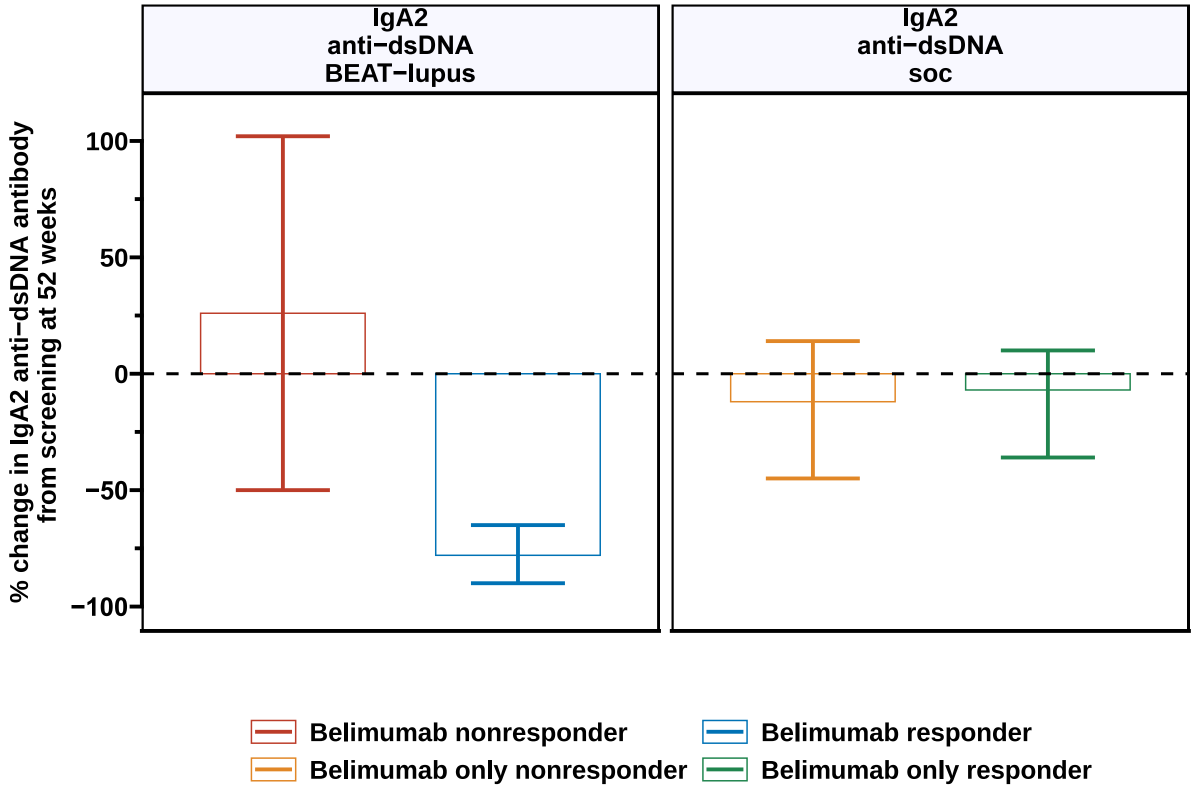

Supplementary Figure 9 | Percentage change in serum IgA2 antibody levels in patients treated with belimumab after rituximab (BEAT-lupus trial), and a separate patient cohort treated with belimumab alone as part of their standard of care (soc), stratified according to clinical response. Percentage change was calculated from screening to 52 weeks in samples from the BEAT-LUPUS trial. For the belimumab alone cohort, the percentage change was calculated from pre-belimumab administration to the time point when the second sample was taken (median interval 18 months, range from 5-25 months). Mean changes with 95% confidence interval are shown.

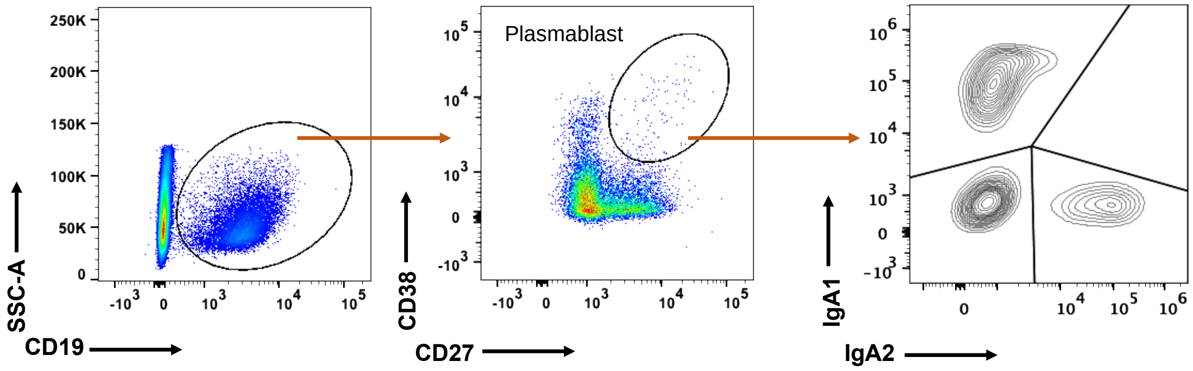

Supplementary Figure 10 | Flow cytometry gating strategy for detecting circulating IgA1 and IgA2 secreting plasmablasts. B cells within PBMC were selected based on CD19 expression. The plasmablast population was defined as $CD19^{+}CD27^{hi}CD38^{hi}$. Anti-IgA1 and IgA2 intracellular staining was used to identify IgA1 and IgA2 secreting plasmablasts.

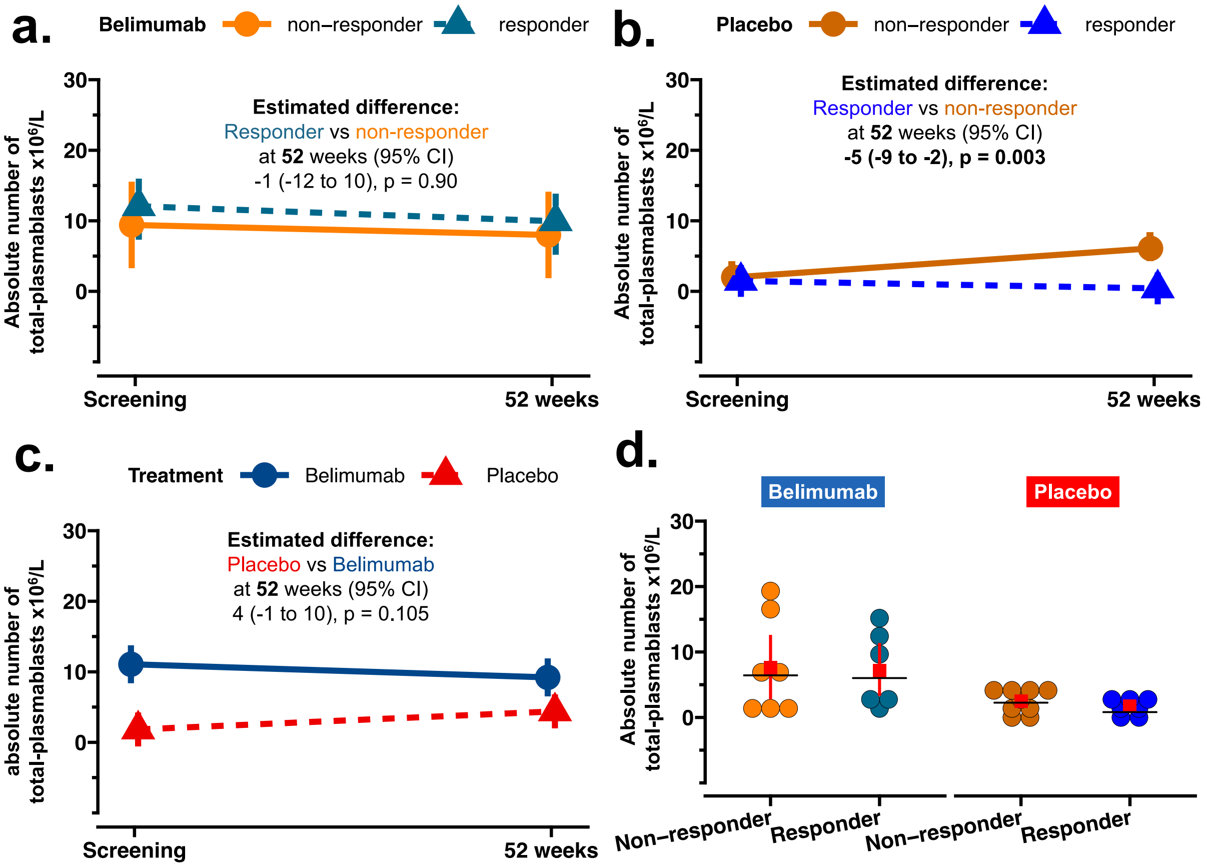

Supplementary Figure 11 a-d | The change in the number of total plasmablasts in peripheral blood during the trial. The absolute number of total plasmablasts at screening and 52 weeks stratified by- **(a)** responders (n=7) and non-responders (n=7) in the belimumab arm, **(b)** responders (n=7) and non-responders (n=9) in the placebo arm, and **(c)** belimumab versus placebo, both after rituximab. A linear regression analysis of covariance model was fitted and adjusted for baseline values, age, gender, concomitant mycophenolate (yes or no), and prednisolone dose at the two time points to calculate expected difference at 52 weeks. Estimated mean with 95% confidence intervals are shown with p value at weeks 52. **(d)** Comparison between the number of total plasmablasts at screening categorized according to clinical response at 52 weeks to belimumab and placebo. Non-parametric pairwise comparison using Dunn's multiple comparison test with Bonferroni's adjustment was adopted to compare between the groups (p values are not shown as not significant).

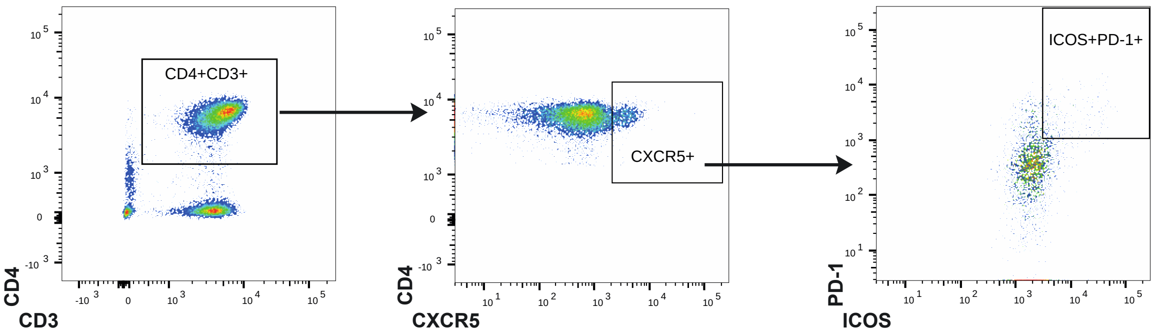

Supplementary Figure 12 | Gating strategy for T-follicular helper cells (defined as $CD4^{+}CXCR5^{+}ICOS^{+}PD1^{+}$).

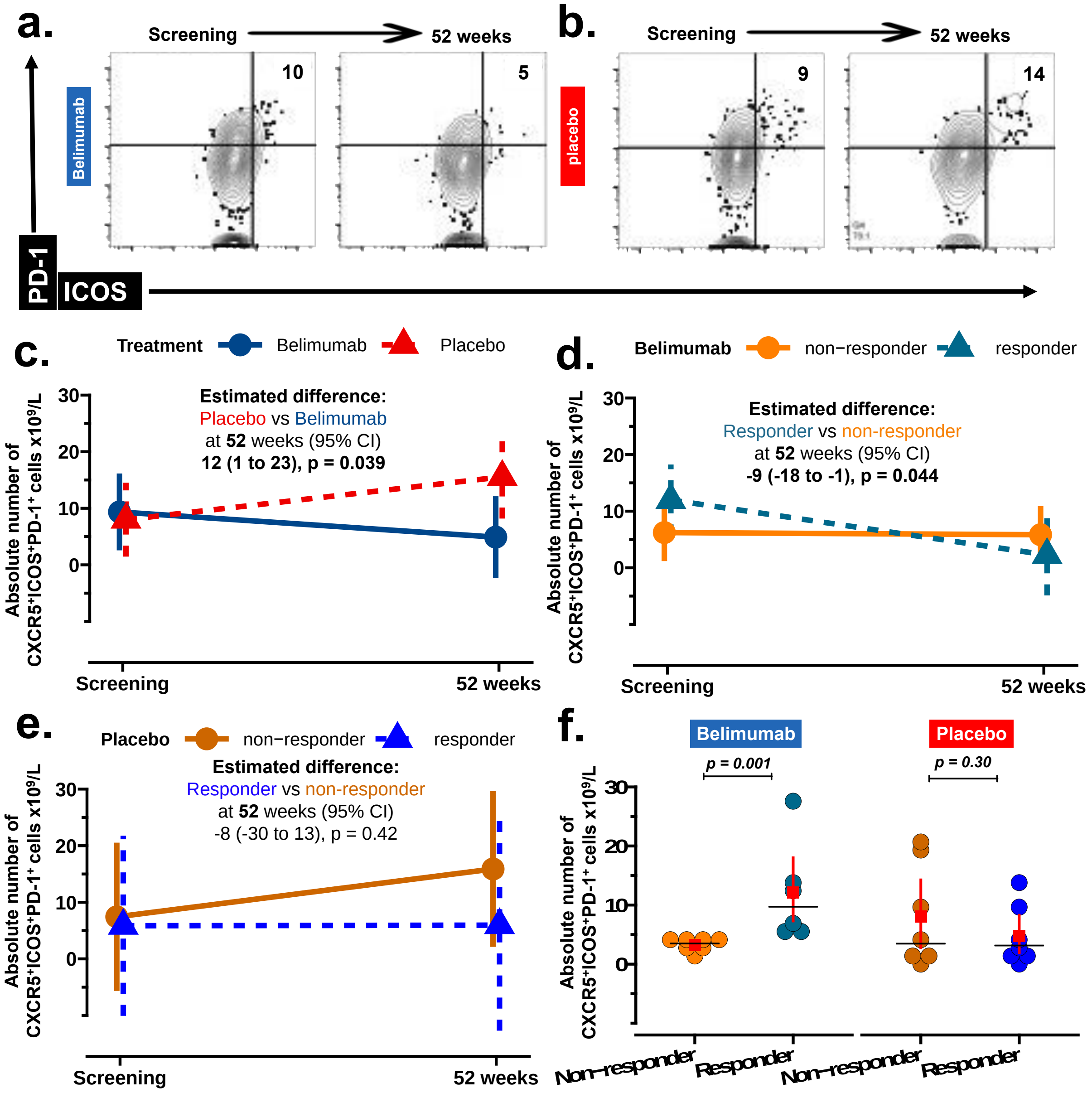

Supplementary Figure 13a-f | Increased baseline T-follicular helper cells predicted clinical response to belimumab after rituximab and fell with treatment. Representative flow cytometry plots of T-follicular helper cells (Tfh, defined as CD4$^+$CXCR5$^+$ICOS$^+$PD-1$^+$) at screening and 52 weeks stratified by **(a)** belimumab (after rituximab) and **(b)** placebo (after rituximab). Cumulative data of the absolute number of Tfh at screening and 52 weeks stratified by- **(c)** belimumab versus placebo, both after rituximab, **(d)** responders (n=7) and non-responders (n=7) in the belimumab arm, and **(e)** responders (n=7) and non-responders (n=9) in the placebo arm. A linear regression analysis of covariance model was fitted and adjusted for baseline values, age, gender, concomitant mycophenolate (yes or no), and prednisolone dose at the two time points to calculate expected difference at 52 weeks. Estimated mean with 95% confidence intervals are shown with p value at weeks 52 is provided. **(f)** Comparison of absolute number of Tfh at screening categorized according to clinical response at 52 weeks to belimumab and placebo. p values are shown above by non-parametric pairwise comparison by Dunn's multiple comparison test with Bonferroni's adjustment.

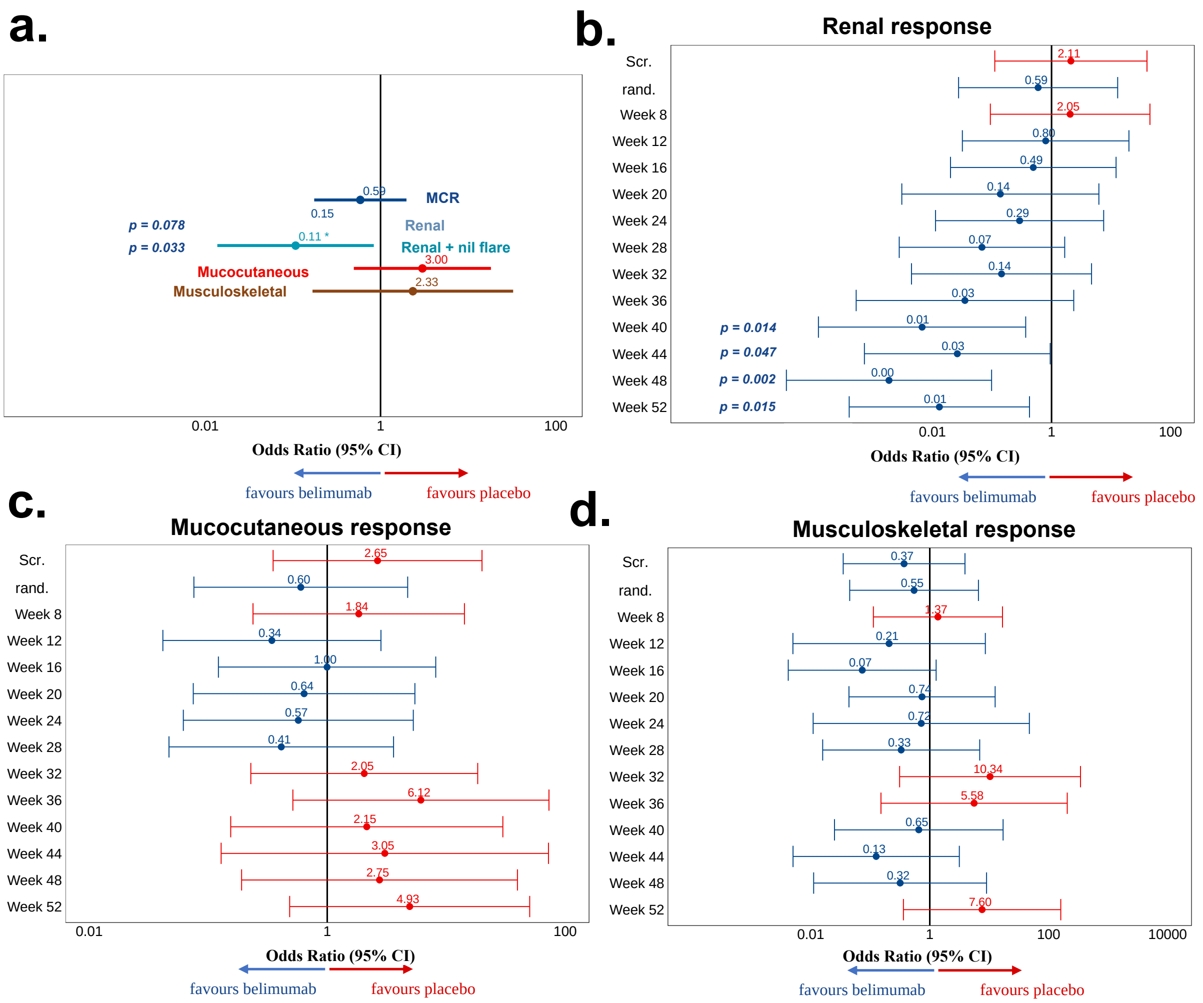

Supplementary Figure 14a-d | Response from baseline through to 52 weeks to belimumab (after rituximab) and placebo (after rituximab) therapy stratified by organ involvement. (a) Odds ratio (OR) with 95% confidence interval (95% CI) of major clinical response (MCR)[$], renal response[†], renal response with no new renal BILAG-2004 A/B flare, mucocutaneous response[††], and musculoskeletal response[†††] at 52 weeks to belimumab (blue favours belimumab response) and placebo (red favours placebo response), by unadjusted univariable logistic regression. OR of either BILAG-2004 (British Isles lupus assessment group-2004 index) A/B in placebo versus belimumab with 95% CI in **(b)** renal domain, **(c)** mucocutaneous domain, and **(d)** musculoskeletal domain from randomisation (rand.) through to 52 weeks. An unadjusted generalised linear mixed-effect model was applied to estimated OR with fixed effect of treatment group or treatment response intercepting with trial times, and random effect of within-patient.

[$] **MCR** - was defined as reduction in BILAG–2004 index A/B scores to BILAG–2004 C/D or remain E in all domains, a reduction in steroid dose to ≤ 7.5mg daily and a modified SLEDAI (Systemic lupus erythematosus disease activity index 2000) –2K score ≤ 2 (without including the anti-dsDNA antibody component) (26).[†] **Renal Response** was defined as no BILAG–2004 index A/B scores in the renal domain, steroid dose ≤ 7.5mg daily, urine protein-creatinine ratio (uPCR) ≤ 50mg/mmol or urine albumin-creatinine ratio (uACR) ≤ 50mg/mmol, no active urine-sediment, and estimated Glomerular Filtration Rate (eGFR) ≥ 60mls/min/1.72m^2 or if eGFR ≤ 60mls/min at baseline, has not fallen by ≥ 20% (27, 28). . [††] **Mucocutaneous and** [†††] **musculoskeletal response -** were defined as no BILAG–2004 A/B in mucocutaneous or musculoskeletal domains, steroid dose ≤ 7.5mg daily, and modified SLEDAI–2K ≤ 2 (without anti-dsDNA antibody component), respectively.

Supplementary Table 5 | Baseline (at screening[†]) variables used in the prediction model for active organ involvement[¶] | Number of samples = 52

Demographics	Age[$], gender[††], race (Caucasian, black, and others)[††], active smoking[††]
Disease status	Disease duration at screening[$], previous rituximab[††], SLEDAI-2K[‡] (systemic lupus erythematosus disease activity index 2000), Numerical[#]-BILAG-2004 (British Isles lupus assessment group – 2004)[‡] (21).
Concomitant treatment	Prednisolone[††], Prednisolone dose[‡], mycophenolate[††], azathioprine[††], methotrexate[††]
Biochemical and immunological profile	Lymphocyte[‡‡], neutrophil[‡], eosinophil[‡‡], basophil[‡‡], monocyte[‡‡], platelet[‡], CD19 counts[‡‡]; c-reactive protein (CRP)[‡‡], erythrocyte sedimentation rate (ESR)[‡], complement C3 (normal or low)[††], urine protein creatinine ratio (uPCR)[‡‡]
Serum autoantibodies	IgG anti-SM[††], IgG anti-Ro[††], IgG anti-LA[††], IgG Anti-RNP[††]<br>IgA1 anti-SM[††], IgA1 anti-Ro[††], IgA1 anti-LA[††], IgA1 Anti-RNP[††]<br>IgA2 anti-SM[††], IgA2 anti-Ro[††], IgA2 anti-LA[††], IgA2 Anti-RNP[††]<br>IgG anti-dsDNA[‡], IgM anti-dsDNA[‡], IgA anti-dsDNA[‡],<br>IgE anti-dsDNA[‡‡‡], IgG1 anti-dsDNA[‡‡‡], IgG2 anti-dsDNA[‡‡‡], IgG3 anti-dsDNA[‡‡‡], IgA1 anti-dsDNA[‡‡‡], IgA2 anti-dsDNA[‡‡‡]<br>*(IgG4 anti-dsDNA antibody was also measured but the values were not above those from healthy donors)*
Serum total Immunoglobulins (Ig)	IgG[‡], IgG1[‡], IgG2[‡], IgG3[‡], IgM[‡], IgA[‡], IgA1[‡], IgA2[‡]
Serum Cytokines	BAFF[‡] (B cell activating factor) *(10)*,<br>IL (interleukin)-6[‡‡] *(11)*, IL-10[‡‡] *(11)*, IL-12[‡‡] *(4)*, IL-17[‡‡] *(11)*,<br>TNF (tumour necrosis factor)-α[‡‡] *(11)*, IFN (Interferon)-α[‡‡] *(2)*, IFN-γ[‡‡] *(11)*
Interferon score and BAFF RNA expression	BAFF[‡] *(14)*, Type I IFN score (IFN-I)[‡] *(14)*, Type I A IFN score (IFN-I A)[‡] *(14)*, Type B IFN total score (IFN-I B)[‡] *(14)*

[†] *Screening refers to the first screening visit before rituximab, randomisation (week 0) occurred 4-8 weeks after screening.*

[¶] ***Active renal disease** defined as – BILAG-2004 (British Isles lupus assessment group – 2004) index A or B in the renal domain at screening with a 24-hour urinary protein > 500mg/day, or urine protein-creatinine ratio (uPCR) > 50mg/mmol, or urine albumin-creatinine ratio (uACR) > 50mg/mmol at baseline, or active urinary sediment with uPCR > 25mg/mmol with active urinary sediment. **Active mucocutaneous** disease defined as either A or B scores in BILAG-2004 mucocutaneous domain. **Active musculoskeletal** disease defied as either A or B scores in BILAG-2004 musculoskeletal domain.*

[††] *Categorial variable.*

[#] ***Numerical-BILAG-2004** - where BILAG-2004 (British Isles lupus assessment group – 2004) A score = 12, B score = 6, C score = 1, D/E score =0.*

Unit changes for the continuous variables for the logistic regression:

[$] *per 1 year change,* [‡] *per 1 unit change,* [‡‡] *log-transformed,* [‡‡‡] *per 1 arbitrary unit change*

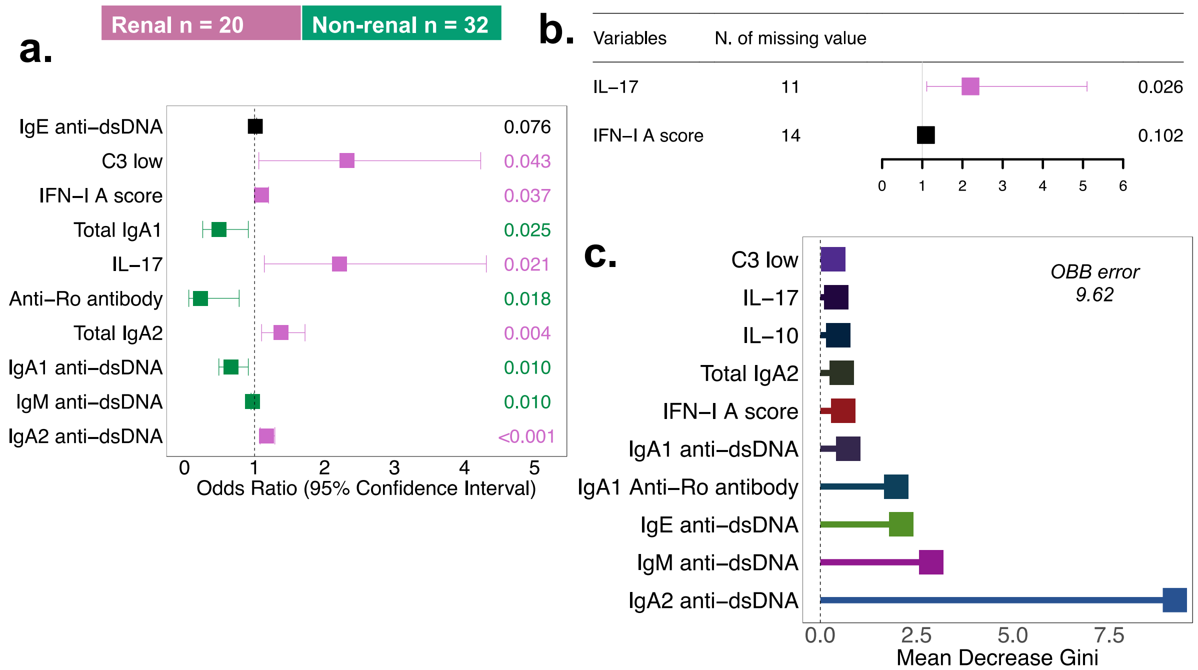

Supplementary Figure 15a-c | Supportive analysis of the parameters to predict active renal disease at screening. (a) Forest plot - odds ratios (OR) with 95% confidence by univariate logistic regression[†] of the top 10-parameters selected by Sparse Partial Least Squares Discriminant Analysis (sPLS-DA). **(b)** univariate logistic regression (complete case analysis) of the parameters with missing value at screening among these 10-parameters. **(c)** Regularised Random Forest (RRF) - Top 10 variables by mean decrease in Gini (ranked) represents the importance of each variable to predict active renal disease.

[†] Unit changes for the continuous variables used in the logistic regression are shown in supplementary table 1.
IFN-I A = Type 1 A interferon score, Ig = Immunoglobulin, IL = interleukin, OBB = out of bag error.

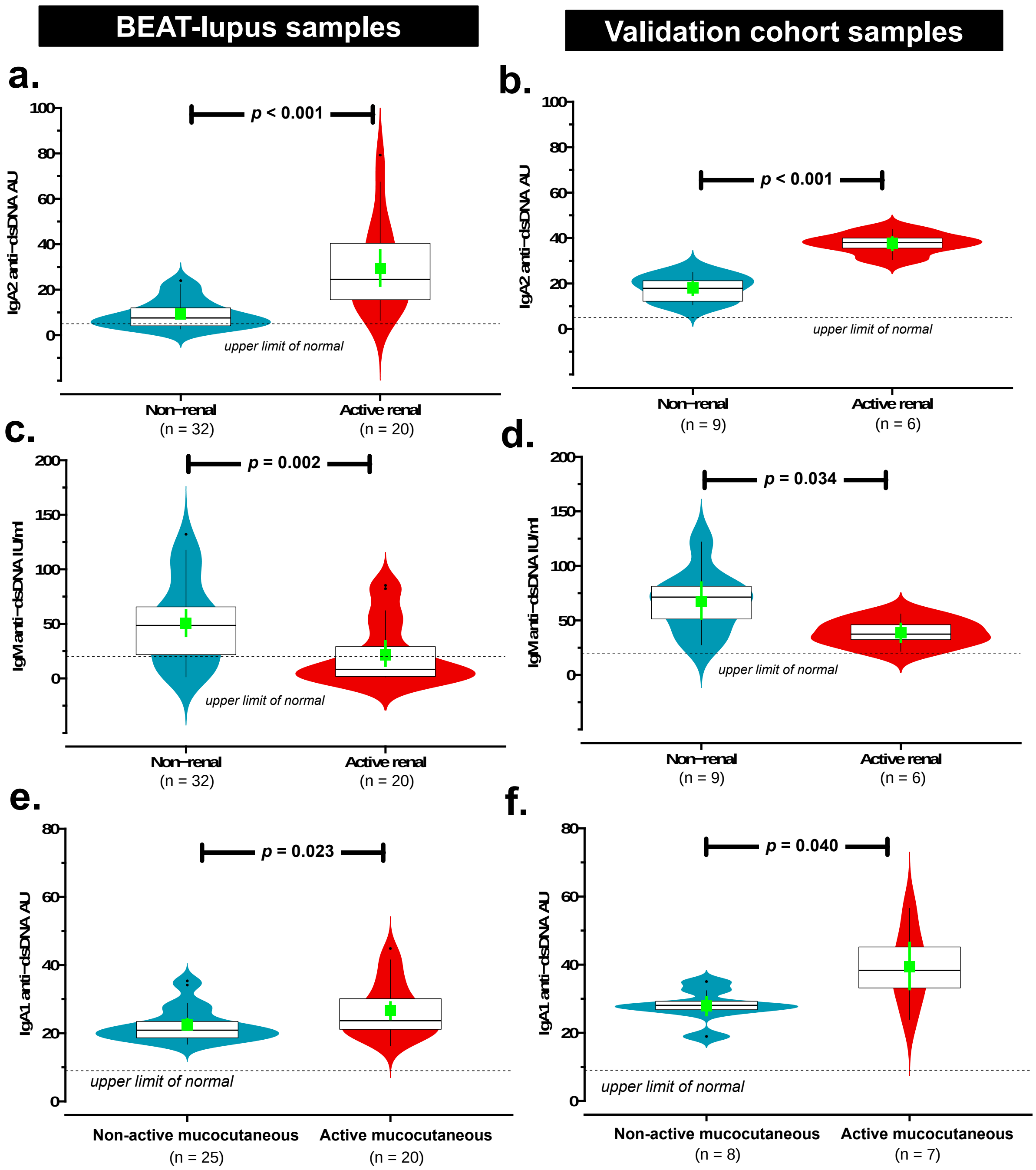

Supplementary Figure 16a-f | Serum IgA2, IgM, and IgA1 anti-dsDNA antibody levels stratified by active renal or mucocutaneus involvement. Serum **(a, b)** IgA2 and **(c, d)** IgM anti-dsDNA antibody levels stratified by active renal disease versus active non-renal disease in patients from the BEAT-LUPUS trial and an independent validation cohort. **(e, f)** Serum IgA1 anti-dsDNA antibody stratified by active mucocutaneous disease versus active non-mucocutaneous disease in patients from the BEAT-LUPUS trial and an independent validation cohort. P values are shown above by non-parametric Mann-Whitney U test. Dotted line indicates upper limit of normal (3 standard deviation above the mean of healthy donor samples). Green box and line indicates mean with 95% confidence interval.

Supplementary Table 6 | Glomerular deposition of IgA1 and IgA2 in lupus nephritis.

Patient	IgA1: Intensity of staining†	IgA1: Distribution	IgA2: Intensity of staining	IgA2: Distribution
Patient_1	3^+	Mesangial	3^+	Mesangial
Patient_2	2^+	Mesangial & sub-epithelial	Negative	N/A
Patient_3	3^+	Mesangial	3^+	Mesangial
Patient_4	3+	Mesangial	3^+	Mesangial
Patient_5	3^+	Mesangial & subepithelial	2^+	Mesangial & segmental

† The intensity of immunohistological deposits in the glomerulus was semi-quantitatively scored using a scale from 0 to 3^+: 0 = none, 1^+ = mild, 2^+ = moderate, 3^+ = intense.

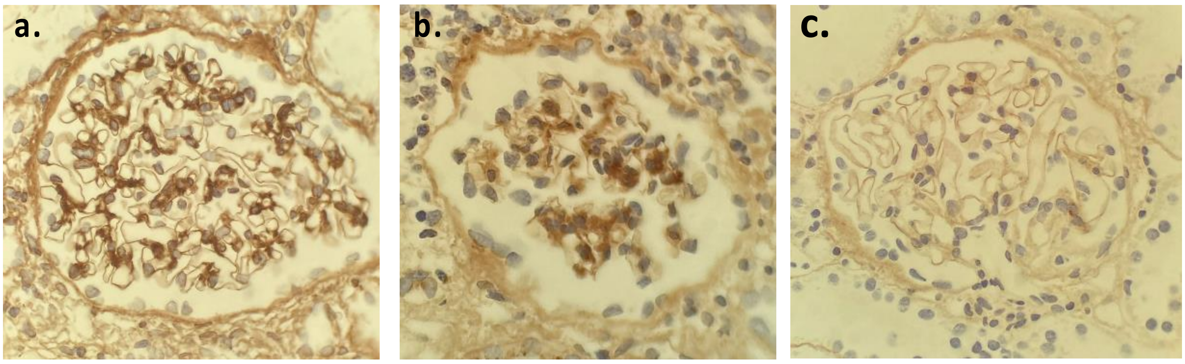

Supplementary Figure 17a-c | Deposition of IgA1 and IgA2 in the glomerulus of a patient with lupus nephritis. (a) IgA1 and (b) IgA2 mesangial deposition (Patient 3 in Supplementary Table 6) with (c) negative control in paraffin-embedded specimens of formalin-fixed renal biopsy tissue (Original magnification x60).

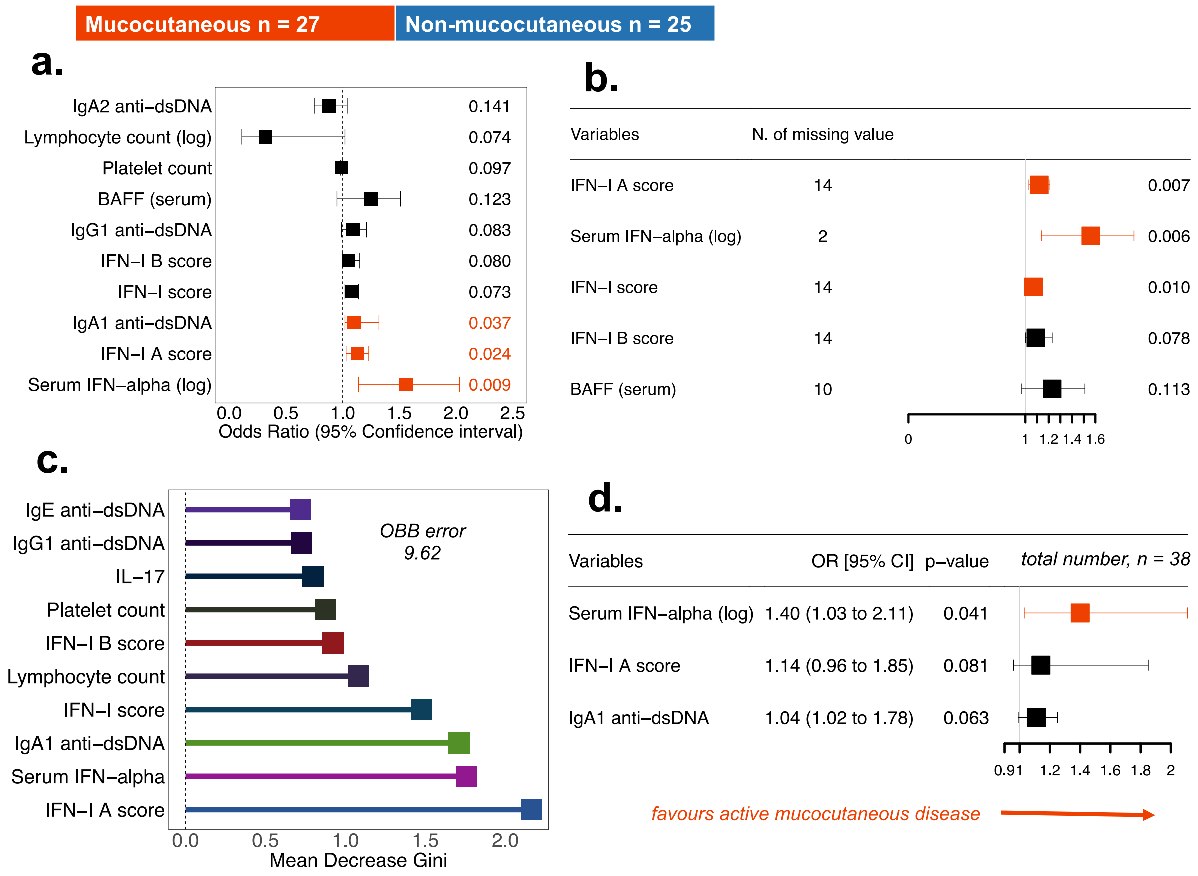

Supplementary Figure 18a-d | Supportive analysis of the parameters to predict active mucocutaneous disease at screening. (a) Forest plot - odds ratios (OR) with 95% confidence by univariate logistic regression† of the top 10-parameters selected by Sparse Partial Least Squares Discriminant Analysis (sPLS-DA). **(b)** univariate logistic regression† (complete case analysis) of the parameters with missing value at screening among these 10-parameters. **(c)** Regularised Random Forest (RRF) - Top 10 variables by mean decrease in Gini (ranked) represents the importance of each variable to predict active renal disease. **(d)** multivariate logistic regression† (complete case analysis) of the 3-parameters chosen by random forest classification algorithm.

† Unit changes for the continuous variables used in the logistic regression are shown in supplementary table 1. BAFF = B-cell activating factor, IFN-alpha = serum interferon-alpha, IFN-I = Type 1 interferon score, IFN-I A = Type 1 A interferon score, IFN-I B = Type 1 B interferon score, Ig = Immunoglobulin, IL = interleukin, OBB = out of bag error.

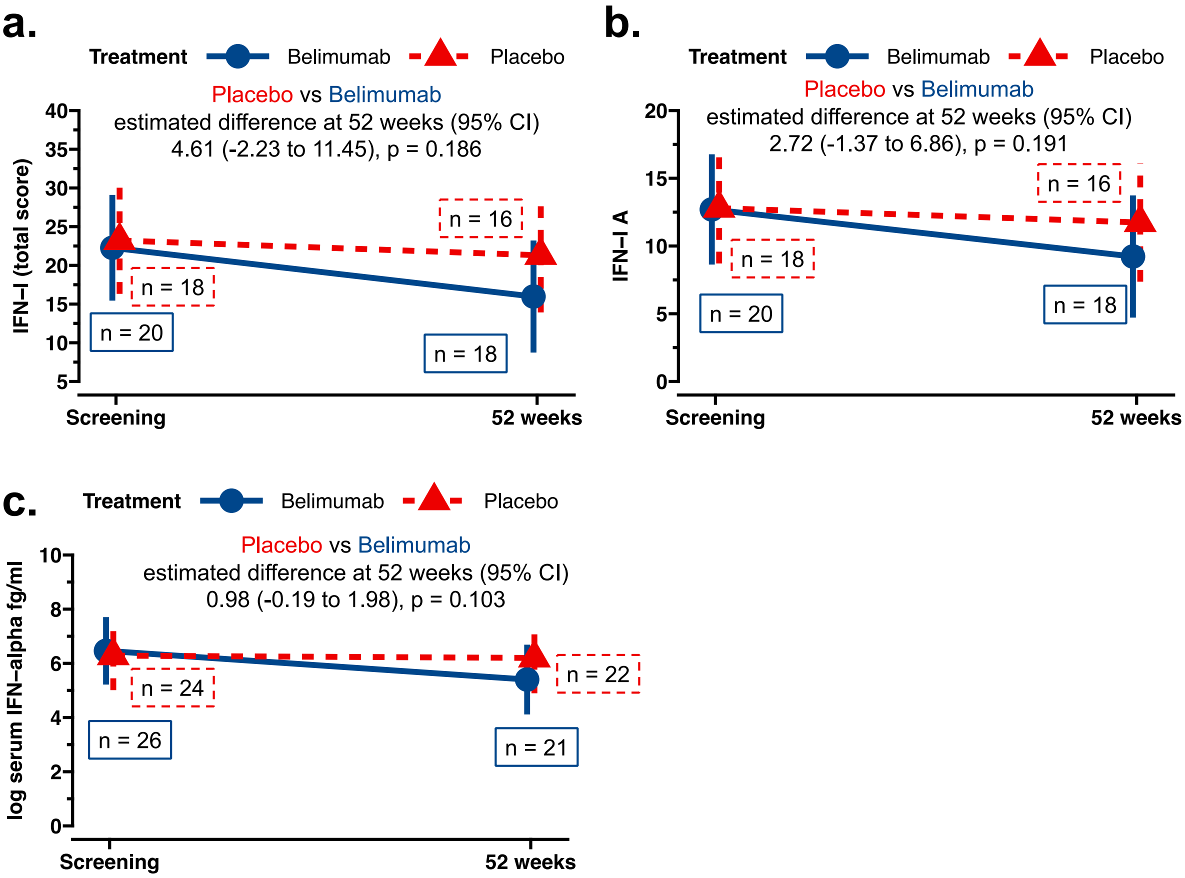

Supplementary Figure 19a-c | (a) Interferon (IFN) type I score (total), (b) type I A score, and (c) serum IFN-α levels (log-transformed) from screening to 52 weeks stratified by treatment: belimumab or placebo (after rituximab). A linear regression analysis of covariance model was fitted and adjusted for baseline values, age, gender, concomitant mycophenolate (yes or no), and prednisolone dose at the two time points to calculate expected difference at 52 weeks. Estimated mean with 95% confidence intervals and number of patients at each time points (n) are shown, and the p values at 52 weeks are provided.

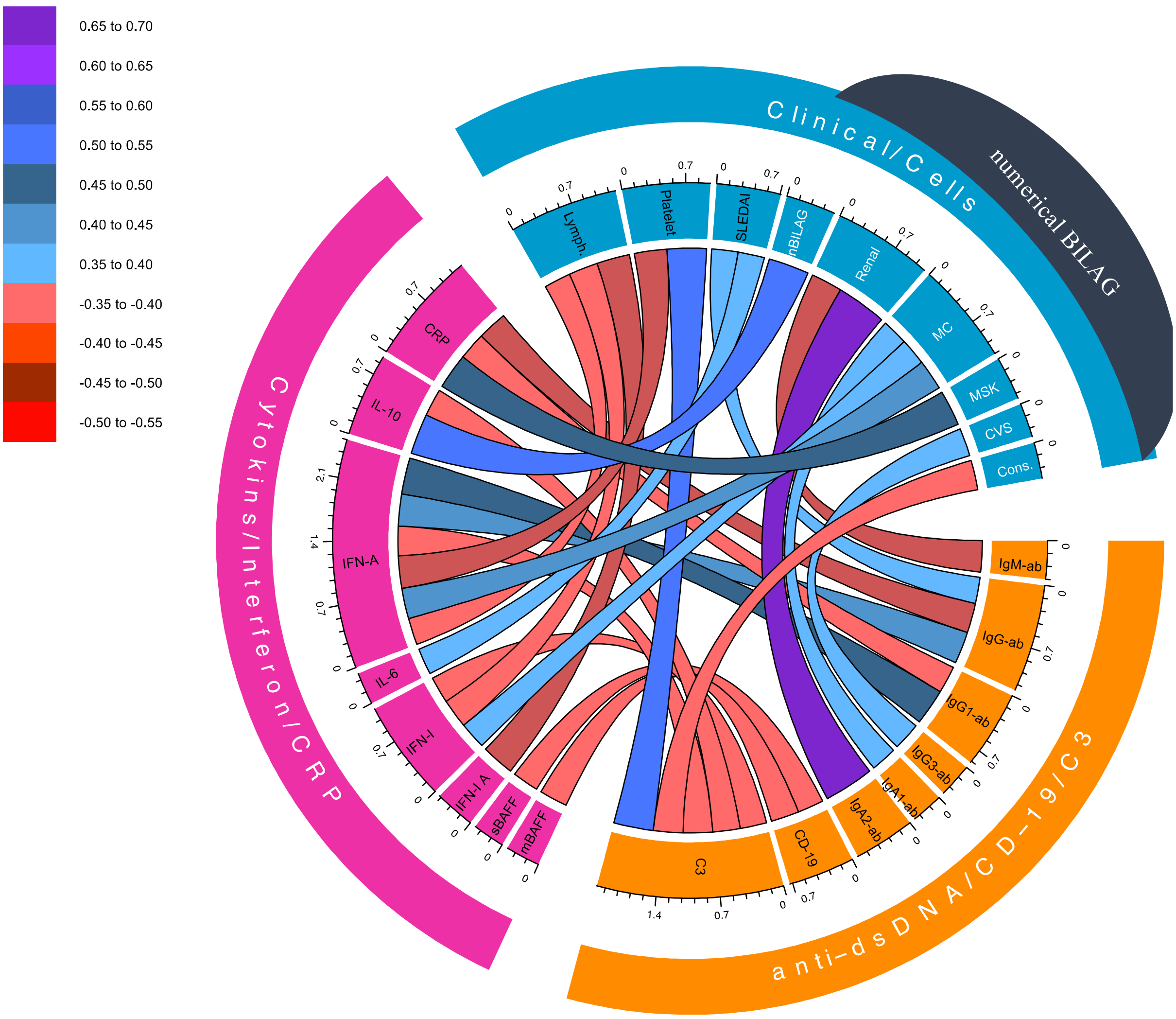

Supplementary Figure 20 | Circos network plot with Spearman's correlations to illustrate interrelationships between clinical and laboratory data at baseline (screening). Correlations were tested among (1) (blue) disease activities score [total and organ specific numerical-BILAG-2004†, and SLEDAI-2K (systemic lupus erythematosus disease activity index 2000)] or cellular components related to disease activities (Lymph = Lymphocyte and platelets), (2) (orange) various subtypes, and subclasses of anti-dsDNA antibodies, CD-19, & C3, and (3) (maroon) cytokines including interferon, interferon scores and C-reactive protein (CRP). Correlations with only p value of ≤ 0.05 are shown. Thickness and colour of the lines show the strength of the Spearman correlation as shown in the legend.

† Numerical-BILAG-2004 denotes to numerical BILAG-2004 (British Isles lupus assessment group – 2004), where BILAG-2004 A score = 12, B score = 8, C score = 1 and D/E score = 0.

ab =Anti-dsDNA antibody, Cons. = Constitutional symptoms, CRP = C-reactive protein, CVS = Cardiovascular, IFN-A = serum interferon-α, IFN-G =serum interferon-γ, IFN-I score = Type I IFN total score, IFN-I A score = Type I A IFN score, Ig = Immunoglobulin, IL = Interleukins, Lymph. = Lymphocyte, mBAFF = B-cell activating factor mRNA expression, MSK = Musculoskeletal, MC = Mucocutaneous, sBAFF = serum B-cell activating factor.

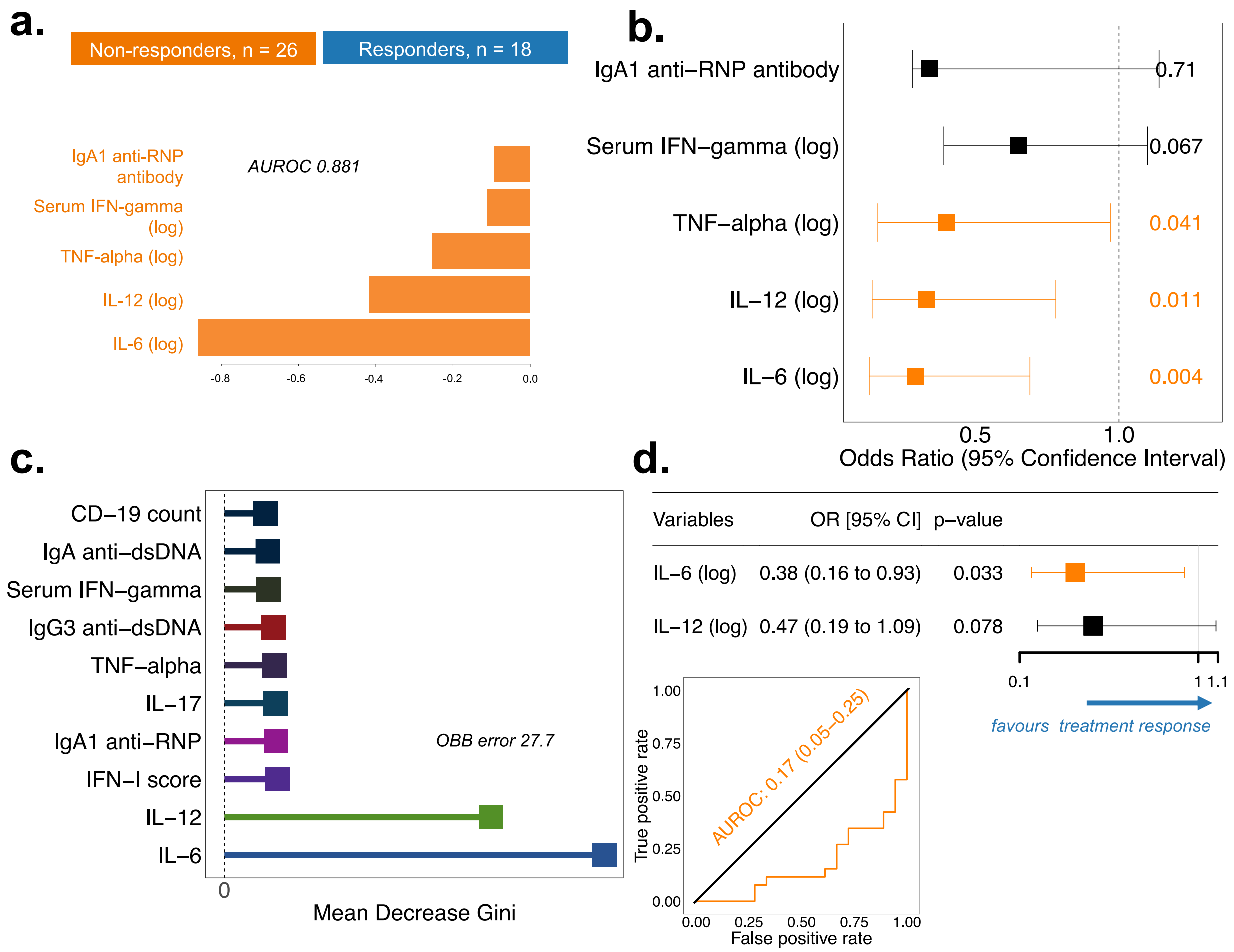

Supplementary Figure 21a-d | Predictors of major clinical response irrespective of trial treatment at 52 weeks. (a) Sparse Partial Least Squares Discriminant Analysis (sPLS-DA) – Factor-loading weights in component 1 are shown for the top 5 (chosen by model optimisation) ranked parameters to predict response irrespective of treatment at 52 weeks. **(b)** Forest plot - odds ratios (OR) with 95% confidence (by univariate logistic regression†) of the predictors chosen by sPLS-DA with p values. **(c)** Regularised Random Forest (RRF) - Top 10 variables by mean decrease in Gini (ranked) represents the importance of each variable to predict treatment response. **(d)** Multiple logistic regression† to construct the final model to predict response at 52 weeks, where variables were selected by random forest classification algorithm; with Area under the Receiver operator characteristic (ROC) curve (AUROC) of this final model to predict response.

† Unit changes for the continuous variables used in the logistic regression are shown in supplementary table 1.

BAFF = B-cell activating factor, OBB = out of bag error, IFN-I score = Type I IFN total score, Ig = immunoglobulin, IL = interleukin, RNP= Ribonucleoprotein, TNF = Tumour necrosis factor.

Supplementary Table 7 | Logistic regression of the top 5-parameters to predict response† irrespective of treatment at 52 weeks (complete case analysis)

Parameters	Number of total patients/ numbers of missing values	Odds ratio†† (95% confidence interval) Univariate model	*p-value*	Odds ratio†† (95% confidence interval) Multivariate model (total number, n = 38)	*p-value*
**IL – 6 (log)**	40/4	0.30 (0.12 to 0.73)	0.008	0.40 (0.15 to 0.97)	0.047
**IL -12 (log)**	40/4	0.37 (0.16 to 0.87)	0.022	0.57 (0.22 to 1.17)	0.141
**TNF - alpha**	40/4	0.42 (0.16 to 1.07)	0.069		
**Serum IFN - gamma**	40/4	0.66 (0.38 to 1.17)	0.155		

† Major clinical response - reduction in BILAG-2004 (British Isles lupus assessment group – 2004) index A or B scores to BILAG-2004 C (or D) in all domains, a reduction in steroid dose to ≤ 7.5mg daily and a modified SLEDAI-2K (systemic lupus erythematosus disease activity index 2000) score ≤ 2 (without anti-dsDNA antibody component).

†† Unit changes for the continuous variables used in the logistic regression are shown in supplementary table 1.

IFN = Interferon, IFN-I score = Type 1 interferon score, IL = interleukin, TNF = Tumour necrosis factor.

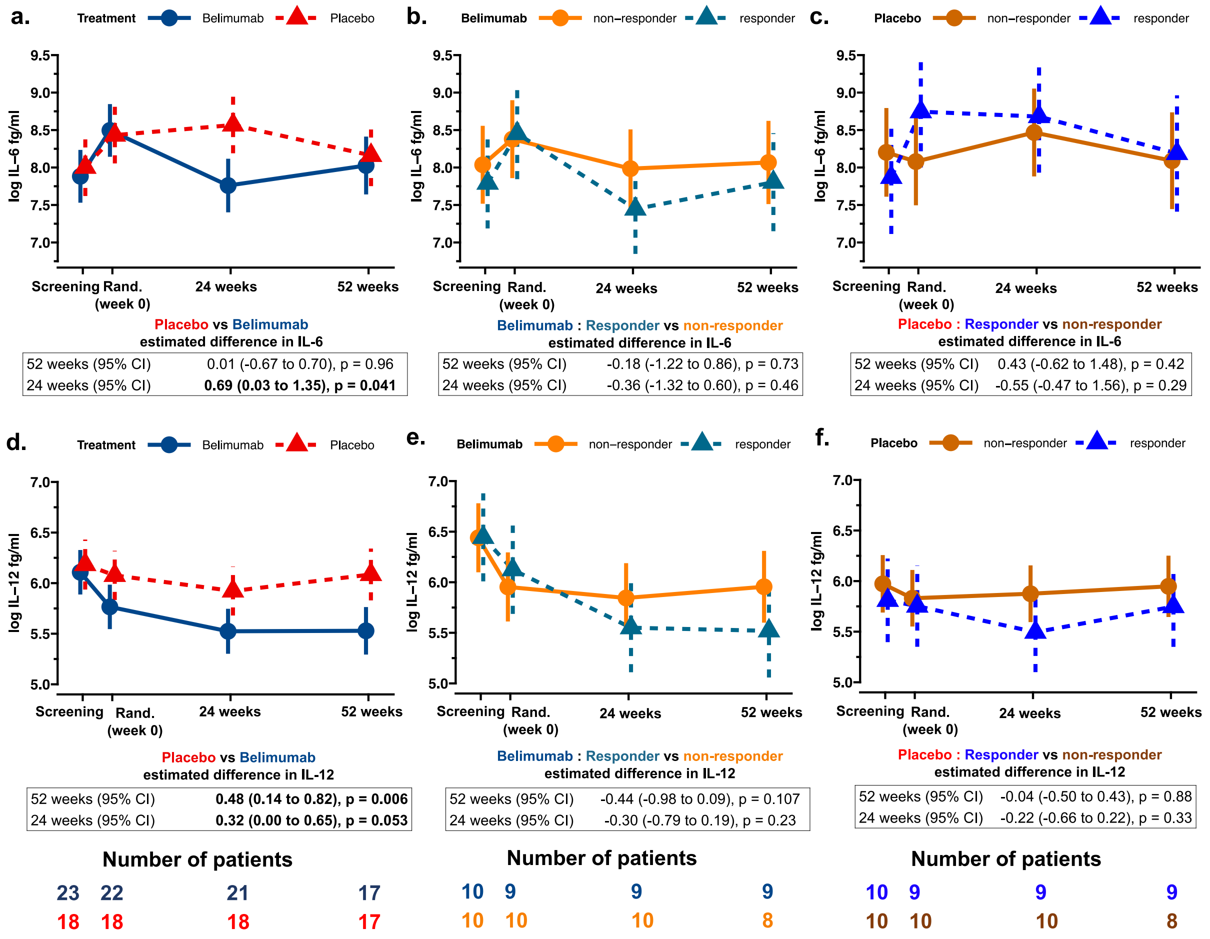

Supplementary Figure 22a-f | Serum IL-6 and IL-12 levels between screening and 52 weeks stratified by trial treatment and response. Changes in serum IL-6 from screening to 52 weeks stratified by - **(a)** treatment with belimumab or placebo (after rituximab), **(b)** responders and non-responders in the belimumab arm, and **(c)** responders and non-responders in the placebo arm. Changes in serum IL-12 from screening to 52 weeks stratified by - **(d)** treatment with belimumab or placebo (after rituximab), **(e)** responders and non-responders in the belimumab arm, and **(f)** responders and non-responders in the placebo arm. A longitudinal linear mixed-effect model was fitted with random patient effect to account for clustering by patients and fixed effect of treatment group intercepting with trial times and adjusted for screening value, age, gender, concomitant mycophenolate (yes or no), and prednisolone dose at respective time points to calculate expected difference at 24 and 52 weeks. Estimated mean with 95% confidence intervals and number of patients at each time points (n) are shown; p values at weeks 24 and 52 are provided.

IL = interleukins, Ig = immunoglobulin, TNF = Tumour necrosis factor.

References:

1. Rice GI, Kasher PR, Forte GM, Mannion NM, Greenwood SM, Szynkiewicz M, et al. Mutations in ADAR1 cause Aicardi-Goutières syndrome associated with a type I interferon signature. Nat Genet. 2012;44(11):1243-8.
2. Lambers WM, de Leeuw K, Doornbos-van der Meer B, Diercks GFH, Bootsma H, Westra J. Interferon score is increased in incomplete systemic lupus erythematosus and correlates with myxovirus-resistance protein A in blood and skin. Arthritis Research & Therapy. 2019;21(1):260.
3. El-Sherbiny YM, Psarras A, Md Yusof MY, Hensor EMA, Tooze R, Doody G, et al. A novel two-score system for interferon status segregates autoimmune diseases and correlates with clinical features. Sci Rep. 2018;8(1):5793.
4. Kyu SY, Kobie J, Yang H, Zand MS, Topham DJ, Quataert SA, et al. Frequencies of human influenza-specific antibody secreting cells or plasmablasts post vaccination from fresh and frozen peripheral blood mononuclear cells. Journal of Immunological Methods. 2009;340(1):42-7.
5. Kourou K, Exarchos TP, Exarchos KP, Karamouzis MV, Fotiadis DI. Machine learning applications in cancer prognosis and prediction. Computational and Structural Biotechnology Journal. 2015;13:8-17.
6. Schaefer J, Lehne M, Schepers J, Prasser F, Thun S. The use of machine learning in rare diseases: a scoping review. Orphanet J Rare Dis. 2020;15(1):145.
7. Robinson GA, Peng J, Dönnes P, Coelewij L, Naja M, Radziszewska A, et al. Disease-associated and patient-specific immune cell signatures in juvenile-onset systemic lupus erythematosus: patient stratification using a machine-learning approach. Lancet Rheumatol. 2020;2(8):e485-e96.
8. Martin-Gutierrez L, Peng J, Thompson NL, Robinson GA, Naja M, Peckham H, et al. Stratification of Patients With Sjögren's Syndrome and Patients With Systemic Lupus Erythematosus According to Two Shared Immune Cell Signatures, With Potential Therapeutic Implications. Arthritis & Rheumatology. 2021;73(9):1626-37.
9. Chicco D, Jurman G. The advantages of the Matthews correlation coefficient (MCC) over F1 score and accuracy in binary classification evaluation. BMC Genomics. 2020;21(1):6.
10. Chung D, Keles S. Sparse partial least squares classification for high dimensional data. Stat Appl Genet Mol Biol. 2010;9(1):Article17.
11. Izquierdo-Verdiguier E, Zurita-Milla R. An evaluation of Guided Regularized Random Forest for classification and regression tasks in remote sensing. International Journal of Applied Earth Observation and Geoinformation. 2020;88:102051.
12. Rohart F, Gautier B, Singh A, Lê Cao K-A. mixOmics: An R package for 'omics feature selection and multiple data integration. PLOS Computational Biology. 2017;13(11):e1005752.
13. Wilkinson L. ggplot2: Elegant Graphics for Data Analysis by WICKHAM, H. Biometrics. 2011;67(2):678-9.
14. Deng H. Guided Random Forest in the RRF Package. ArXiv, abs/1306.0237 [online]. 2013.
15. Kuhn M. Building Predictive Models in R Using the caret Package. Journal of Statistical Software. 2008;28(5):26.
16. Degenhardt F, Seifert S, Szymczak S. Evaluation of variable selection methods for random forests and omics data sets. Brief Bioinform. 2017;20(2):492-503.
17. Kursa MB, Rudnicki WR. Feature Selection with the Boruta Package. Journal of Statistical Software. 2010;36(11):1 - 13.
18. Thiele C. cutpointr: Determine and Evaluate Optimal Cutpoints in Binary Classification Tasks. R package version 1032. 2020.
19. Liu X. Classification accuracy and cut point selection. Stat Med. 2012;31(23):2676-86.

20. Douglas Bates MM, Ben Bolker, Steve Walker. Fitting Linear Mixed-Effects Models Using lme4. Journal of Statistical Software. 2015;67(1):1-48.
21. Yee CS, Cresswell L, Farewell V, Rahman A, Teh LS, Griffiths B, et al. Numerical scoring for the BILAG-2004 index. Rheumatology (Oxford). 2010;49(9):1665-9.
22. Gu Z, Gu L, Eils R, Schlesner M, Brors B. circlize implements and enhances circular visualization in R. Bioinformatics. 2014;30(19):2811-2.
23. White IR, Royston P, Wood AM. Multiple imputation using chained equations: Issues and guidance for practice. Stat Med. 2011;30(4):377-99.
24. van Buuren S, Groothuis-Oudshoorn K. mice: Multivariate Imputation by Chained Equations in R. Journal of Statistical Software. 2011;45(3):1 - 67.
25. Gladman DD, Ibañez D, Urowitz MB. Systemic lupus erythematosus disease activity index 2000. J Rheumatol. 2002;29(2):288-91.
26. McDonald S, Yiu S, Su L, Gordon C, Truman M, Lisk L, et al. Predictors of treatment response in a lupus nephritis population: lessons from the Aspreva Lupus Management Study (ALMS) trial. Lupus Science & Medicine. 2022;9(1):e000584.
27. Davies JC, Midgley A, Carlsson E, Donohue S, Bruce IN, Beresford MW, et al. Urine and serum S100A8/A9 and S100A12 associate with active lupus nephritis and may predict response to rituximab treatment. RMD Open. 2020;6(2).
28. Merrill JT, Neuwelt CM, Wallace DJ, Shanahan JC, Latinis KM, Oates JC, et al. Efficacy and safety of rituximab in moderately-to-severely active systemic lupus erythematosus: the randomized, double-blind, phase II/III systemic lupus erythematosus evaluation of rituximab trial. Arthritis Rheum. 2010;62(1):222-33.
